# QTL identification, fine mapping, and marker development for breeding peanut (*Arachis hypogaea* L.) resistant to bacterial wilt

**DOI:** 10.1007/s00122-022-04033-y

**Published:** 2022-01-20

**Authors:** Feiyan Qi, Ziqi Sun, Hua Liu, Zheng Zheng, Li Qin, Lei Shi, Qingzheng Chen, Haidong Liu, Xiufang Lin, Lijuan Miao, Mengdi Tian, Xiao Wang, Bingyan Huang, Wenzhao Dong, Xinyou Zhang

**Affiliations:** 1 Henan Academy of Crop Molecular Breeding, Henan Academy of Agricultural Science/Key Laboratory of Oil Crops in Huang-Huai-Hai Plains, Ministry of Agriculture/Henan Provincial Key Laboratory for Oil Crop Improvement, Zhengzhou, 450002 Henan China; 2 Hezhou Academy of Agricultural Science, Hezhou, 542899 Guangxi China

## Abstract

**Key message:**

A major QTL, *qBWA12*, was fine mapped to a 216.68 kb physical region, and A12.4097252 was identified as a useful KASP marker for breeding peanut varieties resistant to bacterial wilt.

**Abstract:**

Bacterial wilt, caused by *Ralstonia solanacearum*, is a major disease detrimental to peanut production in China. Breeding disease-resistant peanut varieties is the most economical and effective way to prevent the disease and yield loss. Fine mapping the QTLs for bacterial wilt resistance is critical for the marker-assisted breeding of disease-resistant varieties. A recombinant inbred population comprising 521 lines was used to construct a high-density genetic linkage map and to identify QTLs for bacterial wilt resistance following restriction-site-associated DNA sequencing. The genetic map, which included 5120 SNP markers, covered a length of 3179 cM with an average marker distance of 0.6 cM. Four QTLs for bacterial wilt resistance were mapped on four chromosomes. One major QTL, *qBWA12*, with LOD score of 32.8–66.0 and PVE of 31.2–44.8%, was stably detected in all four development stages investigated over the 3 trial years. Additionally, *qBWA12* spanned a 2.7 cM region, corresponding to approximately 0.4 Mb and was fine mapped to a 216.7 kb region by applying KASP markers that were polymorphic between the two parents based on whole-genome resequencing data. In a large collection of breeding and germplasm lines, it was proved that KASP marker A12.4097252 can be applied for the marker-assisted breeding to develop peanut varieties resistant to bacterial wilt. Of the 19 candidate genes in the region covered by *qBWA12*, nine NBS-LRR genes should be further investigated regarding their potential contribution to the resistance of peanut against bacterial wilt.

**Supplementary Information:**

The online version contains supplementary material available at 10.1007/s00122-022-04033-y.

## Introduction

Cultivated peanut (*Arachis hypogaea* L.) is one of the most important oil crops; its worldwide yield (up to 47 million tons) ranked 4th after soybean, canola, and sunflower. The top 10 countries produced 82.5% of the global peanut yield, while most of these countries are in tropical and subtropical areas, where bacterial wilt is a major peanut disease (Xu et al. 2009; Norman et al. 2009). Peanut bacterial wilt is caused by *Ralstonia solanacearum*, which also causes potato brown rot, bacterial wilt in tomato, tobacco, eggplant, and some ornamental plants, and Moko disease in banana (Paudel et al. 2020). *Ralstonia solanacearum* ranks second among the top 10 pathogens in plant pathology because it is widely distributed and is highly pathogenic (Mansfield et al. 2012). Peanut bacterial wilt had been found in most areas of the 13 major peanut-producing provinces in China, and the disease was estimated to affect 800,000 hectares of agricultural land, accounting for nearly 16% of the total agricultural land in the country (Jiang et al. 2017). This disease can lead to 10–20% peanut yield loss, but in severe cases, yield loss may be as high as 100%. In the last decade, bacterial wilt began to spread from Southern China to the cool uplands at higher latitudes in Northern China. Changes in farming systems and soil treatments resulted in minimal improvements in controlling bacterial wilt (Yu 2011; Jiang et al. 2017). Thus, cultivating disease-resistant peanut varieties is the most economical and effective way for preventing bacterial wilt outbreaks.

Marker-assisted selection (MAS; Ashikari and Matsuoka 2006) is used to screen offspring for the wanted genotypes by applying genetically linked markers, which can greatly improve breeding efficiency by minimizing the effort and time required for phenotypic assessments. Linkage analysis and bulked segregate analysis (BSA; Michelmore et al. 1991) are two popular methods to identify quantitative trait loci (QTLs) associated with target traits. Linkage analysis is based on bi-parental populations, and the factors affecting the detection of QTLs include population size, marker density, logarithm of odds (LOD) threshold, phenotypic variance explained (PVE), and genetic distances between QTLs and markers (Li et al. 2010). Bulked segregate analysis enables the rapid identification of markers linked to any specific gene or genomic region using two bulked from a segregating population with contrasting phenotypic traits (Michelmore et al. 1991). With the rapid development of sequencing technologies, high-throughput genotyping methods are increasingly applied to assist in high-resolution genetic mapping.

Some resistant QTLs for peanut bacterial wilt have been identified via linkage analysis and BSA. For example, two major QTLs on linkage groups LG1 and LG10, with PVE of 12–21%, were detected using a linkage map constructed with simple sequence repeat (SSR) and single nucleotide polymorphism (SNP) markers (Zhao et al. 2016). A major and stable QTL for bacterial wilt resistance (*qBWB02.1*) was mapped on chromosome B02 using a high-density SNP-based genetic linkage map with a recombinant inbred line (RIL) population derived from the YZ9012 × Xuzhou68-4 cross (Wang et al. 2018). Luo et al. (2019) selected lines with contrasting phenotypes from the same RIL population and identified a QTL in the same linkage region by BSA. Moreover, two major QTLs were detected on chromosome B02 using linkage mapping and QTL-seq based on a RIL population derived from a cross between peanut cultivars Xuhua13 and Zhonghua6 (Luo et al. 2020). In conclusion, one major QTL on chromosome B02 was repeatedly detected using different methods and populations. Two diagnostic markers were successfully validated in the lines derived from Yuanza9102 but couldn’t distinguish other susceptible and resistant cultivars (Luo et al. 2019).

A RIL population comprising 521 lines derived from the cross of Yuanza9102 × wt09-0023 was used to fine map the QTL for resistance to bacterial wilt of peanut via high-throughput sequencing. The objectives of this study were: (1) to construct a high-density genetic map according to the dRAD sequencing protocol; (2) to map the resistant QTL for peanut bacterial wilt; (3) to fine map the resistant QTL to a smaller marker interval; (4) to develop Kompetitive Allele-Specific PCR (KASP) markers for MAS and validate their efficacy using different panels of peanut germplasms; and (5) to identify candidate genes for bacterial wilt resistance.

## Materials and methods

### Plant materials

A population comprising 521 F_8:10_ RILs was derived from a cross between Yuanza9102 and wt09-0023 in 2010 at the experimental base of the Henan Academy of Agricultural Science, Yuanyang, China. The female and male parents were planted in two flowerpots, respectively, and three seeds were planted in each pot with 30 cm in high and 45 cm in diameter and then the SSD (single seed descent) method was used for generation acceleration. The female parent (Yuanza9102) is a widely grown variety with high and stable resistance to bacterial wilt and was released by the Institute of Industrial Crops, Henan Academy of Agricultural Science in 2002 (Luo et al. 2019). The male parent (wt09-0023) is a runner type peanut variety provided by Kim Moore (AgResearch Consultants Inc., ACI Seeds, USA) and highly susceptible line to bacterial wilt. The RILs were produced by planting offspring in Henan (in the summer season) and Hainan (in the winter season) for accelerating generation advancement through single seed descent. To validate the diagnostic markers, a panel consisting of 71 breeding lines, 47 of which were derived from the crosses involving the resistant parent (Yuanza9102), and another panel comprising 317 peanut germplasm lines were phenotyped and genotyped using the newly developed KASP markers. Additionally, peanut cultivars Zhonghua6 and Zhanghua12, which were released by the Oil Crops Research Institute of the Chinese Academy of Agricultural Sciences, were used as the resistance and susceptibility check varieties, respectively.

### dRAD library construction, sequencing, and SNP calling

Leaf tissue of single F_8_ plants and the two parents were used for DNA isolation. The genomic DNA was first digested with restriction enzyme EcoRI, fragments of 300–500 bp were subsequently collected, amplified, and sequenced on an Illumina HiSeq 4000 platform to generate 150-bp paired-end reads. The sequencing depths for the two parents and the RILs were approximately 25 × and 5 ×, respectively. After filtering the reads to eliminate adapters and low-quality reads, the remaining clean reads were aligned to the reference genome (Bertioli et al. 2019) using the bwa-0.7.10 (Li and Durbin 2019). The uniquely mapped reads were used to call SNPs with the SAMtools v0.1.19 and GATK v3.3.0 programs (McKenna et al. 2010). The homozygous and polymorphic SNPs between the two parents were retained for the RIL population. The SNPs with missing and heterozygosity rates less than 10% and a sequencing depth greater than 3 × were used for the following analysis. The clean data obtained in this study have been submitted to the BioProject database of NCBI (BioProject ID: PRJNA704994).

### Genetic map construction and QTL mapping

The filtered SNPs of the 521 F_8_ lines were used to construct a genetic linkage map. First, the BIN module of QTL IciMapping v4.2 (Meng et al. 2015) was used to delete the completely linked, redundant markers. Next, Joinmap v5.0 (Van Ooijen 2018) was used to identify linkage groups (LGs) based on the independence LOD scores from 2 to 28, and the maximum likelihood algorithm was applied for determining loci orders using Kosambi's mapping function. The genetic map was plotted using the R package LinkageMapView (Ouellette et al. 2018). QTL analysis was performed using multiple QTL mapping (MQM) of MapQTL v6.0 (Van Ooijen 2009), by setting the mapping step size as 0.1 cM and the LOD threshold as 2.5.

### Development of KASP markers for MAS and validation of the markers in broad germplasm collections

The polymorphic SNPs between the two parents (whole-genome resequencing data; approximately 20 × coverage) in the region covered by the putative QTL on LG12 including 1 Mb up- and downstream were converted to KASP markers according Trick et al. (2012). Additionally, 521 RILs, 71 breeding lines, and 317 peanut germplasms were genotyped using the newly developed KASP markers. Phenotypes were generated for all the lines and the marker/trait-association validated to narrow down the candidate gene region. The QTL region of LG12 was drawn with the MapChart v2.3 (Voorrips 2002).

### Phenotyping of bacterial wilt resistance

A disease nursery established in 1980 in Hezhou city, the Guangxi Zhuang Autonomous Region, China, was used to assess bacterial wilt resistance. Because of the limited size of the disease nursery field, the plant material was tested in consecutive trials from 2016 to 2020. The parental lines and 412 lines of the RIL population were assessed in 2016 and 2017 (F_9_ and F_10_ generations), the two parents and the remaining 109 lines of the RIL population (F_10_ generation) and 71 breeding lines were assessed in 2018, and eventually, 317 peanut germplasms were assessed in 2019 and 2020. Twenty seeds of each material were sown in a randomized block design with two replications, and the resistant and susceptible check varieties were included in each block. The number of emerged plants was recorded 10–16 days after sowing. Survival rates were calculated at the seedling (20–30 days after sowing), flowering (31–40 days), pegging (41–60 days), and harvesting (120–130 days) stages. The survival rate was calculated using the following formula: Survival rate (%) = (no. of survived plants/no. of emerged plants) × 100.

### Statistical analysis of phenotyping data

An analysis of variance (ANOVA) among the 412 lines over their survival rates at each of the four different stages was performed in 2016 and 2017 using the AOV module of QTL IciMapping v4.2 (Meng et al. 2015), and broad-sense heritability was estimated accordingly. The distribution of survival rates was visualized in box, and violin plots produced using R v3.5.

## Results

### Phenotypic variations of bacterial wilt resistance

**Fig. 1:**
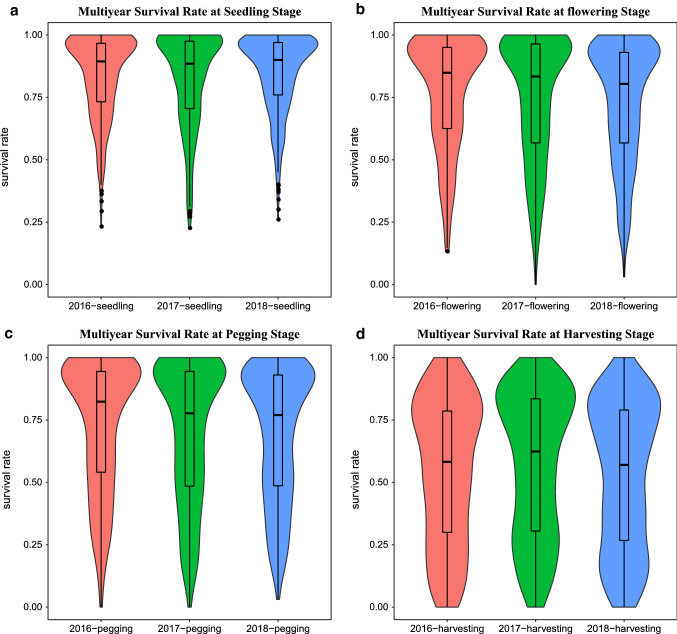
Boxplots and violin plots for the survival rate of RILs at four stages across 3 years. **A** seedling stage; **B** flowering stage; **C** pegging stage; **D** harvesting stage. The survival rate of 412 lines was assessed in 2016 and 2017, and that of all 521 lines was contained in 2018, in which for the survival rate of the 412 lines, the average of the survival rate in 2016 and 2017 was used

The female parent (Yuanza9102) and the male parent (wt09-0023) displayed clear difference in resistance to bacterial wilt at flowering, pegging, and harvesting stages across 3 years (Table S1). Additionally, considerable phenotypic variations in response to bacterial wilt were observed among the RILs at four observed stages across the 3 years (Table S1 and Fig. 1). The survival rates of a subset of 412 lines were investigated in 2016 and 2017, and that of all 521 lines were included in 2018, in which the average of the survival rates of the 412 lines recorded in 2016 and 2017 was used. The kurtosis and skewness values according Table S1 as well as the violin plots in Fig. 1D showed that the survival rates at the harvesting stage were closer to a normal distribution. The ANOVA results indicated a highly significant genotype effect (*G*) at all four stages, but the environment (*E*) and genotype × environment interactions (*G* × *E*) were less significant or not significant (Table S2), the reason may be that the experiment was conducted in a single location. The broad-sense heritability ranged from 0.8 to 0.9 based on entry mean at four stages (Table S2).

### dRAD sequencing and SNP identification

The dRAD sequencing protocol was used for analyzing the two parents and 521 RILs. Approximately 1918 Gb clean data (Q20 > 94%) were generated, including 13.4 billion reads (Table S3). About 116.3 and 132.8 million reads were obtained for the female and male parents, respectively, whereas 10.5–62.1 million reads were obtained for the RILs (Table S3). More than 98% of the reads were mapped to the reference genome (Table S3). A total of 291,195 polymorphic SNPs were identified for the two parents, of which 90,807 were homologous and used to screen for SNPs in the RILs. The resulting 29,301 SNPs were further filtered, after which the 24,852 remaining SNPs were used for the following analysis.

### Construction of the genetic linkage map

**Fig. 2:**
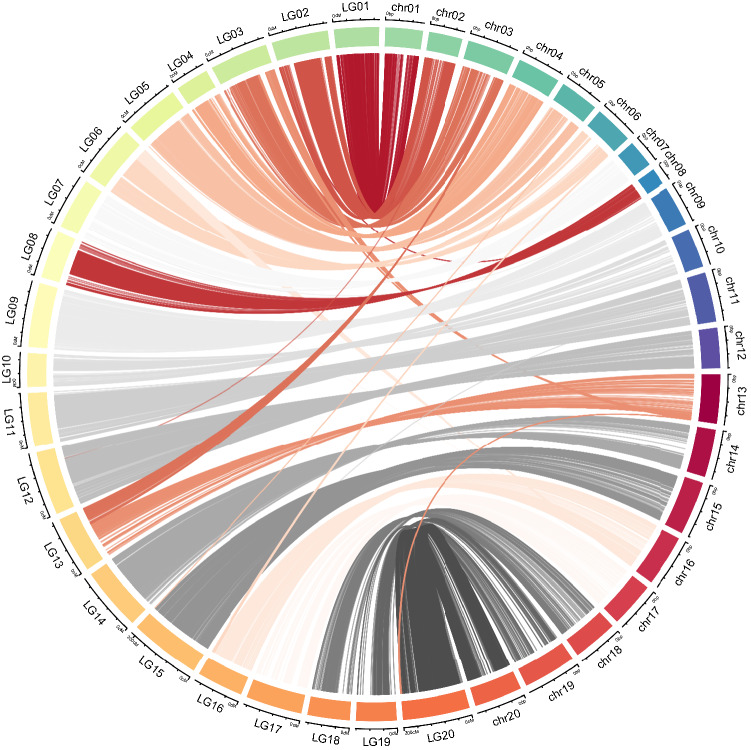
Collinearity of the linkage groups constructed using a RIL population with the reference genome (*Arachis hypogaea*.cv.tifrunner). The prefix LG stands for genetic map on left-hand side whereas, prefix chr stands for corresponding physical map on right-hand side

Redundant markers were removed from the filtered SNP set using the BIN module of QTL IciMapping v4.2, and the remaining 5151 SNPs were used to construct a genetic linkage map based on the 521 lines using Joinmap v5.0. After eliminating 31 redundant SNPs, 5120 SNPs were assigned to 20 LGs (Table 1 and Fig. S1). The whole map length was about 3179 cM, with an average marker distance of 0.6 cM (Table 1). The marker number among linkage groups varied from 114 (LG10) to 479 (LG15), the map length ranged from 100.0 cM (LG04) to 211.3 cM (LG15), and the average marker distance ranged from 0.4 (LG15) to 1.1 cM (LG19) (Table 1). The maximum inter-marker distance was 25.8 cM (LG19), and 96.8% of the inter-marker distances were less than 3.0 cM. The collinearity analysis revealed a high degree of synteny between the genetic linkage map and the corresponding chromosomes of the reference genome, with the exception of some translocations between LG03 and LG13 and between LG06 and LG16 (Fig. 2).

**Table 1:** Summary of the high-density linkage groups (LGs) obtained for the RIL population

Linkage ID	Total marker	Total distance (cM)	Average distance (cM)	Max inter-marker distance (cM)	Inter-markers distances ≤ 3 cM (%)
LG01	228	133.8	0.6	4.6	98.7
LG02	284	170.7	0.6	16.5	98.2
LG03	255	175.6	0.7	20.1	97.7
LG04	158	100.0	0.6	9.9	96.8
LG05	291	162.3	0.6	9.1	99.0
LG06	276	169.6	0.6	8.9	99.3
LG07	203	151.2	0.7	17.0	94.6
LG08	221	144.6	0.7	9.8	95.9
LG09	321	192.1	0.6	4.3	98.1
LG10	114	101.1	0.9	10.8	93.0
LG11	322	159.1	0.5	7.4	98.5
LG12	349	191.7	0.6	3.8	98.6
LG13	209	167.7	0.8	12.6	96.7
LG14	374	188.7	0.5	7.6	98.9
LG15	479	211.3	0.4	4.0	99.6
LG16	210	139.4	0.7	5.5	97.1
LG17	118	168.7	1.4	21.9	89.8
LG18	154	126.5	0.8	17.2	93.5
LG19	115	121.2	1.1	25.8	93.9
LG20	439	204.1	0.5	5.2	98.4
Total	5120	3179.4	0.6	–	96.8

### QTL identification and fine mapping for bacterial wilt resistance

**Fig. 3:**
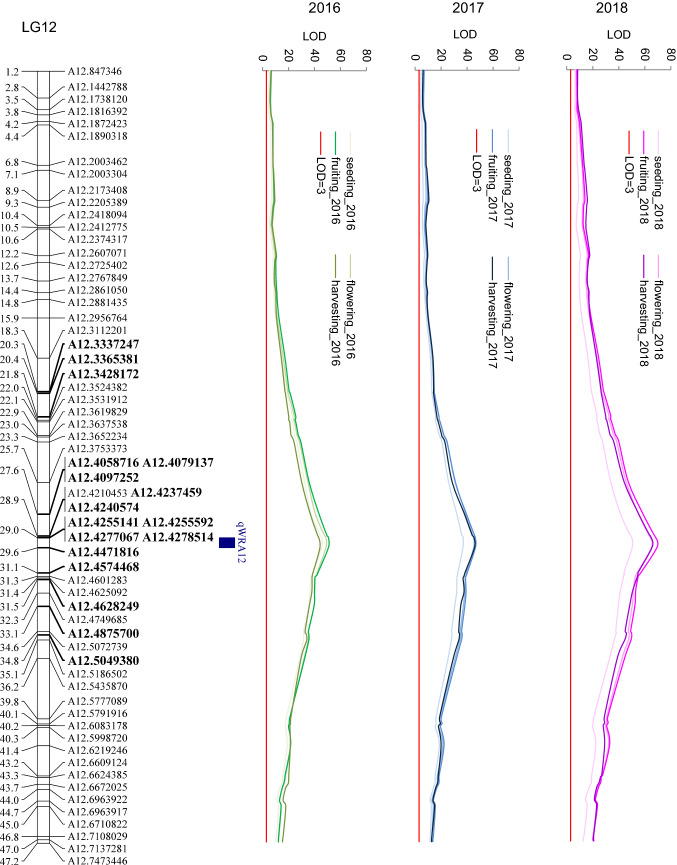
LOD curves and position of the *qBWA12* on LG12 under four stages in 3 years. Only the linkage area close to *qBWA12* is shown

QTL identification for bacterial wilt resistance was performed, respectively, based on survival rates of the 412 RILs at four development stages in 2016 and 2017. In 2018, the average survival rates for the 412 RILs based on the above mentioned 2 years’ data together with the survival rates of the additional 109 lines collected in 2018 were used to identify QTLs. Four QTLs were detected in all 3 years, which were distributed on LG03, 12, 14, and 20, with an LOD score greater than 2.5 (Table 2). A major QTL on LG12 (*qBWA12*) was consistently detected at all four stages in all 3 years, with LOD scores of 32.8–66.0, and with PVE of 31.2–44.8% (Table 2). The additive effect was between 7.9 and 14.1, implying the resistance allele was derived from the female parent Yuanza9102. Moreover, *qBWA12* was located on chromosome 12 and was localized to a 2.7 cm interval (29.1–31.8 cM) flanked by SNP site A12.4210453 and A12.4601283, corresponding to a 390.8 kb physical region. To fine map *qBWA12*, 42 homologous and polymorphic SNPs between the two parents in a 3.3–5.1 Mb physical region of chromosome 12 were selected to convert to KASP markers. Then 17 successfully genotyped KASP markers (Table S4) confirmed polymorphisms between the two parents and were used to genotype the 521 RILs. These markers were integrated in the genetic map of LG12, and a fine mapping QTL analysis for bacterial wilt resistance was performed (Fig. 3). Accordingly, *qBWA12* was fine mapped to a 0.6-cM-interval flanked by A12.4255141 and A12.4471816, and the physical region was shortened to 216.7 kb from 390.8 kb.

**Table 2:** QTLs identified for BW resistance at four stages in 3 years

QTL	LG	Position (cM)	Left–right marker	LOD	PVE (%)	Add effects (%)	Environment
*qBWA03*	LG03	101.7–102.4	A03.128299006–A03.128522169	2.6–5.7	2.5–4.9	2.9–4.8	(2016_harvesting; 2017_fruiting; 2017_harvesting; 2018_flowering; 2018_fruiting; 2018_harvesting)
*qBWA12*	LG12	29.1–31.8	A12.4210453–A12.4601283	32.8–66.0	31.2–44.8	7.9–14.1	(2016_seedling; 2016_flowering; 2016_fruiting; 2016_harvesting; 2017_seedling; 2017_flowering; 2017_fruiting; 2017_harvesting; 2018_seedling; 2018_flowering; 2018_fruiting; 2018_harvesting)
*qBWA14*	LG14	59.4–60.0	A14.9011103–A14.9111536	3.0–4.9	3.2–5.3	-4.8 to -3.9	(2016_harvesting; 2017_fruiting; 2017_harvesting; 2018_harvesting)
*qBWA20*	LG20	182.6–182.8	A20.141889915–A20.141884654	2.5–4.4	2.2–5.0	2.0–4.6	(2016_seeding; 2016_flowering; 2016_harvesting; 2017_seedling; 2017_flowering; 2017_fruiting; 2017_harvesting; 2018_seedling; 2018_flowering; 2018_fruiting; 2018_harvesting)

### KASP marker validation

**Fig. 4:**
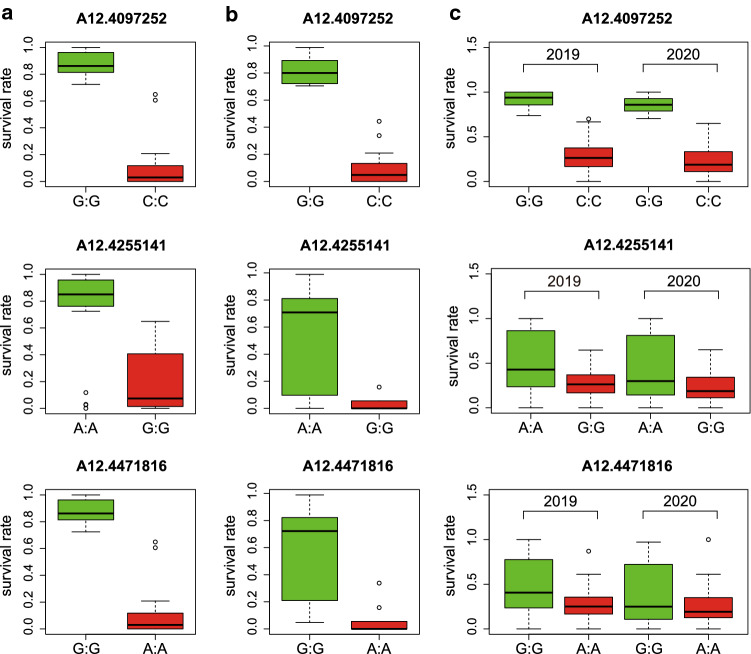
Phenotypic differences between two genotypes at three SNP sites for 71 breeding lines and 317 peanut germplasms lines. **a** Phenotypic differences between two genotypes at three SNP sites for 47 lines derived from Yuanza9102. **b** Phenotypic differences between two genotypes at three SNP sites for 24 breeding lines. **c** Phenotypic differences between two genotypes at three SNP sites for 317 peanut germplasms

A total of 71 breeding lines in 2018 (Table S5) and 317 peanut germplasms in 2019 and 2020 (Table S6) were evaluated for bacterial resistance. The 17 KASP markers successfully genotyped in the RIL population were used to genotype the two panels of peanut material (Figs. 4 and S2–4). For the 47 breeding lines derived from Yuanza9102, significant phenotypic differences were observed between the two genotypes at 14 SNP sites (A12.4058716–A12.5049380) (Fig. S2), including A12.4097252 and the two flanking markers A12.4255141 and A12.4471816 (Fig. 4a). In contrast, for the 24 breeding lines that were not derived from Yuanza9102 as well as the 317 peanut germplasms, significant phenotypic differences were observed between the two genotypes only at A12.4097252 (Fig. 4b, c, Figs. S3–4). These results implicated that A12.4097252 was tightly linked with bacterial wilt resistance, and the resistant allele was G instead of C at the SNP site.

### Potential candidate genes for bacterial wilt resistance

Nineteen candidate genes for bacterial wilt resistance were predicted in the region covered by *qBWA12* on the basis of the annotated reference genome database (Bertioli et al. 2019) (Table 3). Eleven of these candidate genes were nucleotide-binding site-leucine-rich repeat (NBS-LRR) genes, and one gene (*arahy.KAG5PZ*) encoded a disease resistance-related protein with the NB-ARC (nucleotide-binding adapter shared by APAF-1, R proteins, and CED-4) domain along with the LRR (Table 3). To further screen the candidate genes, the whole-genome resequencing reads of Yuanza9102 were aligned to the reference genome, and the mapping rates in the regions covered by the 19 candidate genes were determined (Table 3). Nine genes, with mapping rates of 100%, may be unrelated to bacterial wilt resistance, including two NBS-LRR genes and the NB-ARC-LRR gene. Another nine NBS-LRR genes, with mapping rates of 38.0–99.9%, may play an important role in resistance to bacterial wilt. The alignment of reads to the eight genes with a mapping rate less than 96.0% visualized using the Integrative Genomics Viewer (Robinson et al. 2011) that is presented in Figure S5.

**Table 3:** The predicted candidate genes for BW resistance in the region covered by the major *qBWA12*

ID	gene model	chr	Gene location	annotation	Mapping rate (%)
1	*arahy.AU62Q4*	A12	4209114–4210556(−)	UDP-Glycosyltransferase superfamily protein	100.0
2	*arahy.V6I7WA*	A12	4236131–4239737(+)	Disease resistance protein (NBS-LRR class)	95.2
3	*arahy.45D7QY*	A12	4242720–4243363(−)	Pre-mRNA-splicing factor cwc26	100.0
4	*arahy.TE7I1T*	A12	4257212–4259902(+)	disease resistance Protein (NBS-LRR class)	99.9
5	*arahy.0EHV1A*	A12	4280257–4284123(+)	Disease resistance protein (NBS-LRR class)	93.6
6	*arahy.KAG5PZ*	A12	4292471–4292740(+)	LRR and NB-ARC domain disease resistance protein	100.0
7	*arahy.W2W4JI*	A12	4292896–4294501( +)	Disease resistance protein (NBS-LRR class)	100.0
8	*arahy.P88NCS*	A12	4323774–4327025(+)	Disease resistance protein (NBS-LRR class)	91.6
9	*arahy.HZH8EM*	A12	4339150–4343716(+)	Disease resistance protein (NBS-LRR class)	52.6
10	*arahy.5D95TJ*	A12	4352358–4356221( +)	Disease resistance protein (NBS-LRR class)	89.8
11	*arahy.D89ZXX*	A12	4365013–4366609(+)	Disease resistance protein (NBS-LRR class)	100.0
12	*arahy.WQJN9J*	A12	4394900–4398148(+)	Disease resistance protein (NBS-LRR class)	80.0
13	*arahy.LURW66*	A12	4410277–4414843( +)	Disease resistance protein (NBS-LRR class)	38.0
14	*arahy.MXY2PU*	A12	4423482–4426655(+)	Disease resistance protein (NBS-LRR class)	86.0
15	*arahy.TC7Y0P*	A12	4442239–4445553(−)	WRKY family transcription factor	100.0
16	*arahy.KL1Z6U*	A12	4458290–4461891(−)	Histone superfamily protein	100.0
17	*arahy.G8BSRK*	A12	4463005–4471216(−)	Alpha/beta hydrolase domain-containing protein 11	100.0
18	*arahy.DSQC6N*	A12	4474755–4474964(−)	Uncharacterized protein	100.0
19	*arahy.GPD66T*	A12	4479488–4482537(+)	Pentatricopeptide (PPR) repeat-containing protein	94.2

## Discussion

Cultivated peanut (*Arachis hypogaea* L.) is an allotetraploid species (AABB, 2*n* = 4*x* = 40) with a large genome (approximately 2.7 Gb). It diverged from two diploid ancestral species about 9400 years ago, which resulted in limited genetic diversity (Bertioli et al. 2016). Therefore, the first two generations of molecular markers were not widely used for cultivated peanut (Pandey et al. 2014; Chen et al. 2016). The advance of next-generation sequencing technology and the publication of peanut genomes (Bertioli et al. 2016, 2019; Chen et al. 2019; Zhuang et al. 2019) have accelerated QTL mapping-related research and MAS-based breeding.

### Improved genetic linkage map construction and QTL mapping

The publication of the first peanut reference genome (Bertioli et al. 2016) and the development of second generation sequencing methods greatly increased the efficiency and accuracy of QTL mapping in peanut (Shasidhar et al. 2017; Wang et al. 2018; Han et al. 2018). The first genetic linkage maps were mainly constructed using SSR markers, and these maps were limited to a few hundreds of markers (Huang et al. 2015; Prasad et al. 2015; Tseng et al. 2016; Zhao et al. 2016). Today, most genetic maps are constructed using SNP markers from next generation sequencing, and often, over 2000 SNPs, which are ten times more than formerly used SSR markers (Wang et al. 2018; Gangurde et al. 2020; Li et al. 2019; Liu et al 2020; Zhang et al. 2019), are mapped. Additionally, using the *A. hypogaea* genome as a reference genome substantially increased the mapping rate of sequencing data as well as the number of SNPs. Luo et al. (2019) obtained 122 million homologous and polymorphic SNPs between two parents using the genomes of *A. duranensis* and *A. ipaensis* as reference while 326 million SNPs were called using the genome of *A. hypogaea* cv. Tifrunner as reference with the same sequencing protocol and similar sequencing depth (unpublished data).

In addition to marker density, population size is also one critical factor for resolution and power of QTL mapping (Li et al. 2010). The two RIL populations included in studies by Wang et al. (2018) and Luo et al. (2020) comprised 188 and 268 lines, respectively, and the final physical regions covered by the resistant QTL for peanut bacterial wilt were both ~ 2 Mb. In the current study, the physical interval was narrowed down to about 390.8 kb using 521 lines. Because a larger population size results in more recombinant events, smaller QTL confidence intervals and stronger linkage between QTL and phenotypes can be achieved.

### Collinearity analysis of the genetic linkage and physical maps

An analysis of the LGs and their corresponding chromosomes revealed a relatively high collinearity between the LGs and chromosomes, with the exception of four LGs (Fig. 2). The marker orders in some regions on LG03, LG06, LG13, and LG16 were inconsistent with the corresponding physical orders (Fig. 2) because two reciprocal translocations occurred between the ends of LG03 and LG13 and between the ends of LG06 and LG16. For details, the SNPs from Arahy.13:135672462–145340054 were mapped to the end of LG03, and the SNPs from Arahy.03:133160681–141868592 were grouped at the end of LG13. Similarly, the SNPs from Arahy.16:143810297–150964456 and Arahy.06:108333059–111980823 were mapped at the ends of LG06 and LG16, respectively. There were also some translocations between LG02 and Arahy.08 and between LG20 and Arahy.13. The SNPs from Arahy.08:51599619–51799542 were mapped to the head of LG02, and the SNPs from Arahy.13:145768538–146301976 were mapped to the end of LG20. These translocations resulted from assembly mistakes in the reference genome (*A. hypogaea* cv. Tifrunner version 1). A large portion of the distal end was swapped between Arahy.03 and Arahy.13 and between Arahy.06 and Arahy.16 in the updated genome (*A. hypogaea* cv. Tifrunner version 2; https://www.peanutbase.org/peanut_genome_v1_v2). Hence, the genetic map may provide valuable information for the assembled genome.

### Comparison between linkage mapping and BSA

Linkage mapping and BSA are two widely used methods for identifying QTLs associated with target traits, but their mapping power and resolution are not comparable. For the same RIL population (Yuanza9102 × Xuzhou68-4), a physical interval of approximately 2.3 Mb (chromosome B02:2501128–4797039) covered by *qBWB02.1* was obtained from linkage mapping (Wang et al. 2018), whereas a larger interval (approximately 3.4 Mb) was obtained using the BSA method (Luo et al. 2019). Thus, the mapping resolution of linkage analysis is greater than that of BSA, especially for QTLs with a small PVE. Although, sequencing costs were greatly reduced for BSA, only two bulked pools consisting of a few individuals with extreme phenotypes were sequenced, but the identified QTL had a larger interval, and more makers and recombinants would be needed to fine-map the QTL. In the present study, the identification of QTL *qBWA12* for peanut bacterial wilt spanned a physical region of only 390.8 kb once again demonstrated the high resolution of extensive RIL linkage mapping.

### Predicted candidate genes

Proteins containing NBS-LRRs, which exist in many plants, form the biggest group of R proteins involved in effector-triggered immunity (Jones and Dangl 2006). These proteins can enhance plant resistance to various pathogens. For example, two NBS-LRR genes (*RPS4* and *RRS1*) cloned from *Arabidopsis thaliana* reportedly mediate resistance to several pathogens (e.g., bacterial wilt pathogen) in other transgenic plants, including oilseed rape, tomato, and cucumber (Gassmann et al.^1999^; Deslandes et al. 2002; Vinatzer et al. 2013; Narusaka et al. 2014). Moreover, the expression of a novel peanut NBS-LRR gene (*AhRRS5*) is up-regulated in both resistant and susceptible peanut cultivars in response to *R. solanacearum,* and the overexpression of this gene enhances the resistance of transgenic tobacco plants to *R. solanacearum* (Zhang et al. 2017).

Using the annotated Tifrunner reference genome (Bertioli et al. 2019), 11 NBS-LRR and one NB-ARC-LRR genes were detected in the interval covered by *qBWA12* (Table 3). The clean resequencing reads for the resistant parent (Yuanza9102) were precisely mapped to two NBS-LRR and the NB-ARC-LRR genes, with a mapping rate of 100%, whereas the mapping rates were 38.0–99.9% for nine other NBS-LRR genes. The relatively low mapping rates may be explained by the considerable differences in the gene sequences between Yuanza9102 and Tifrunner. Furthermore, the possible contributions of these nine NBS-LRR genes to the resistance of peanut to bactrial wilt will need to be investigated.

### KASP marker design and validation

Two KASP markers (AraipB02:3740746 and AraipB02:5804063) were designed and validated using the RIL population derived from the Yuanza9102 × Xuzhou68-4 cross (Luo et al. 2019). The data revealed that both markers can only be used for the lines derived from Yuanza9102 and conclude that the resistance allele from Yuanza9102 may differ from the resistance alleles in other resistant lines (Luo et al. 2019). In this study, 17 polymorphic SNPs between the two parents in the target QTL interval, which were detected by analyzing the whole-genome resequencing data, were used to develop KASP markers for fine mapping *qBWA12* and validated using two panels of peanut materials. Among of them, 14 KASP markers, including two markers flanking *qBWA12* (A12.4255141 and A12.4471816), can be used to screen for resistant lines derived from Yuanza9102, while only one (A12.4097252) is applicable for all peanut germplasms. Therefore, A12.4097252 can be applied in marker-assisted breeding to develop peanut varieties resistant to bacterial wilt with various genetic backgrounds, while the other 13 flanking markers could be employed in the screening of the resistant genotypes derived from Yuanza9102.

## Supplementary Information

Below is the link to the electronic supplementary material.

Supplementary file1 (PDF 1256 KB)

Supplementary file2 (PDF 388 KB)

## Data Availability

All data generated or analyzed during this study are included in the manuscript and its Additional file 1 to Additional file 9. The clean resequencing data obtained in this study are available at the BioProject database at NCBI under the BioProject ID: PRJNA704994 (https://dataview.ncbi.nlm.nih.gov/object/PRJNA704994?reviewer=hsjf26260jisd87vn2p091s5u6). Materials used in this study are available from the corresponding authors.
