# Supplementary material for: QTL identification, fine mapping, and marker development for breeding peanut (Arachis hypogaea L.) resistant to bacterial wilt
Source: Theor Appl Genet. 2022 Jan 20;135(4):1319–30. doi: 10.1007/s00122-022-04033-y (PMC9033696; doi:10.1007/s00122-022-04033-y)
Supplement: Supplementary file 1 — Supplementary file1 (PDF 1256 KB) [file 122_2022_4033_MOESM1_ESM.pdf]

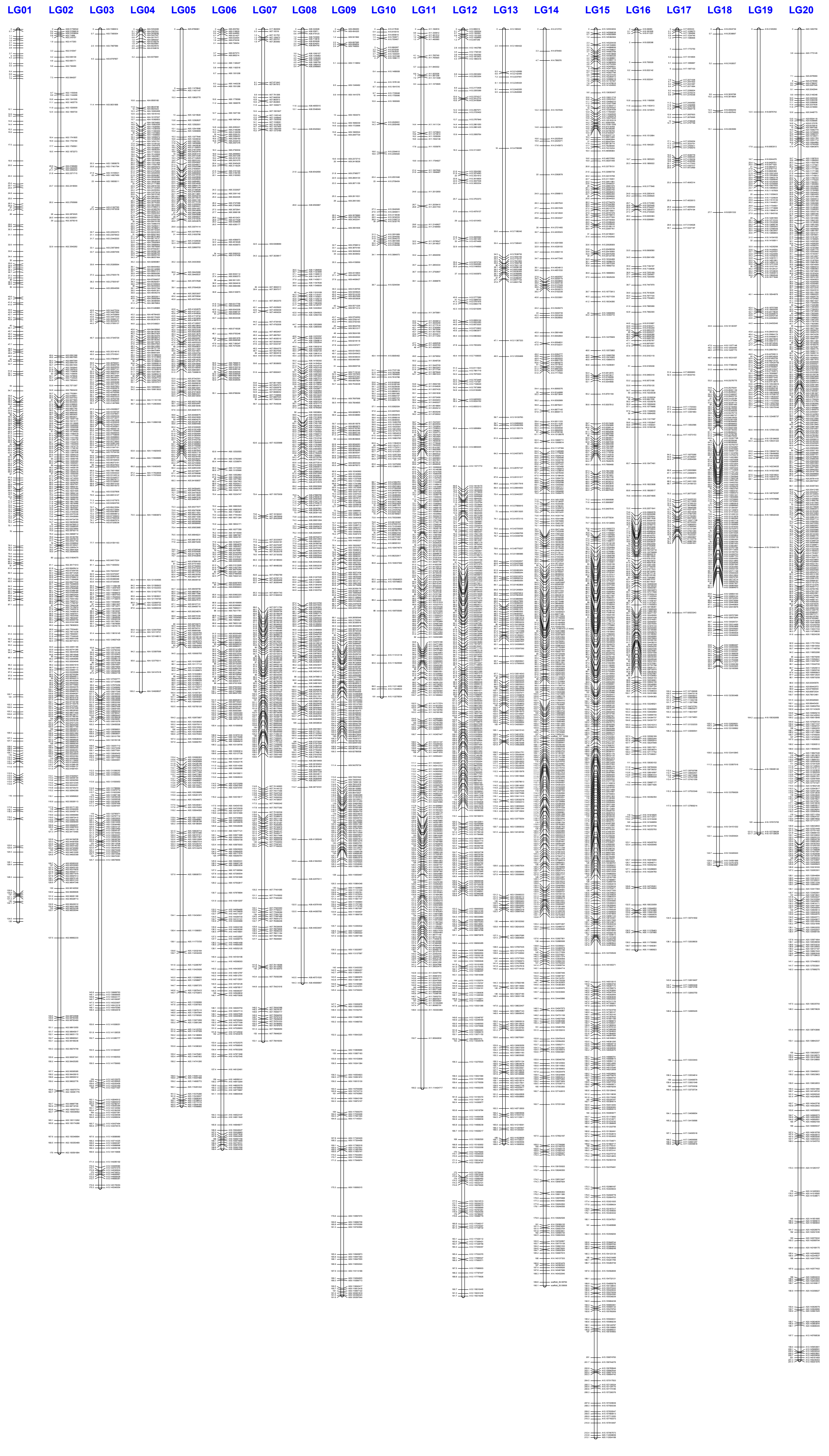

Supplementary Figure 1. Genetic map of the Yuanza9102×wt09-0023 RIL population.

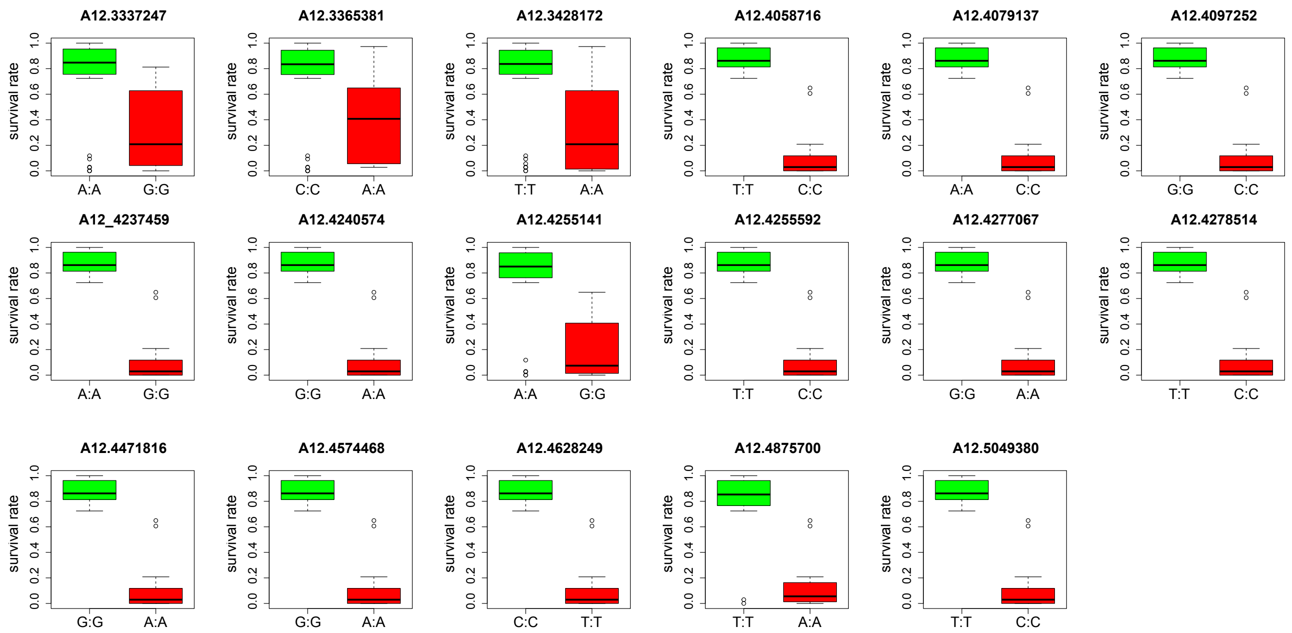

Supplementary Figure 2. Phenotypic differences between the two genotypes at 17 SNP sites for 47 breeding lines derived from Yuanza9102.

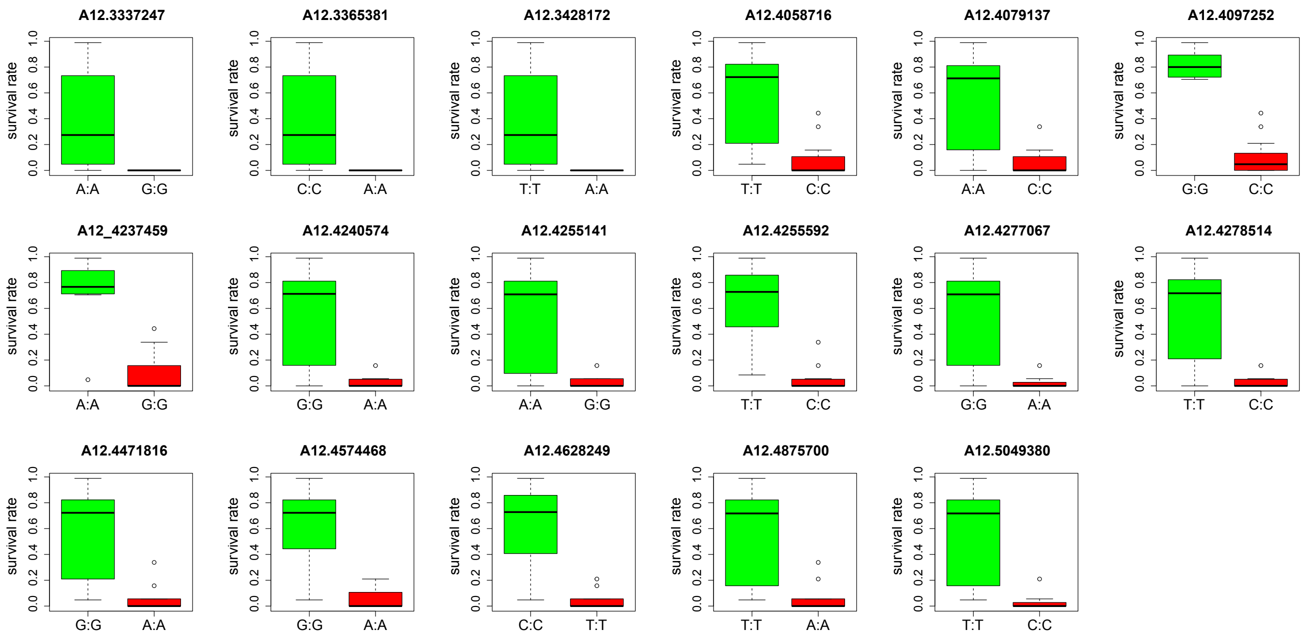

Supplementary Figure 3. Phenotypic differences between the two genotypes at 17 SNP sites for 24 breeding lines.

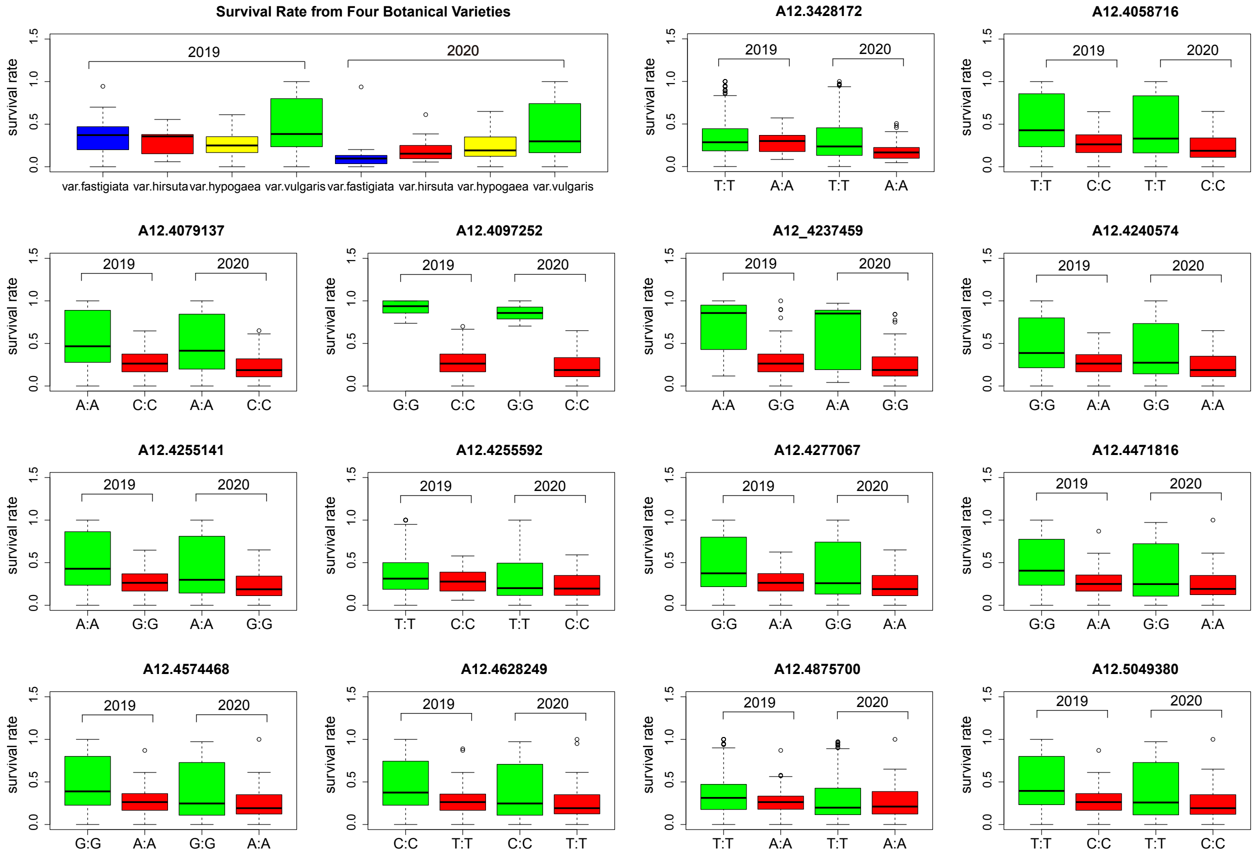

Supplementary Figure 4. Phenotypic differences between four botanical types and between two genotypes at 14 SNP sites for 317 peanut germplasm lines.

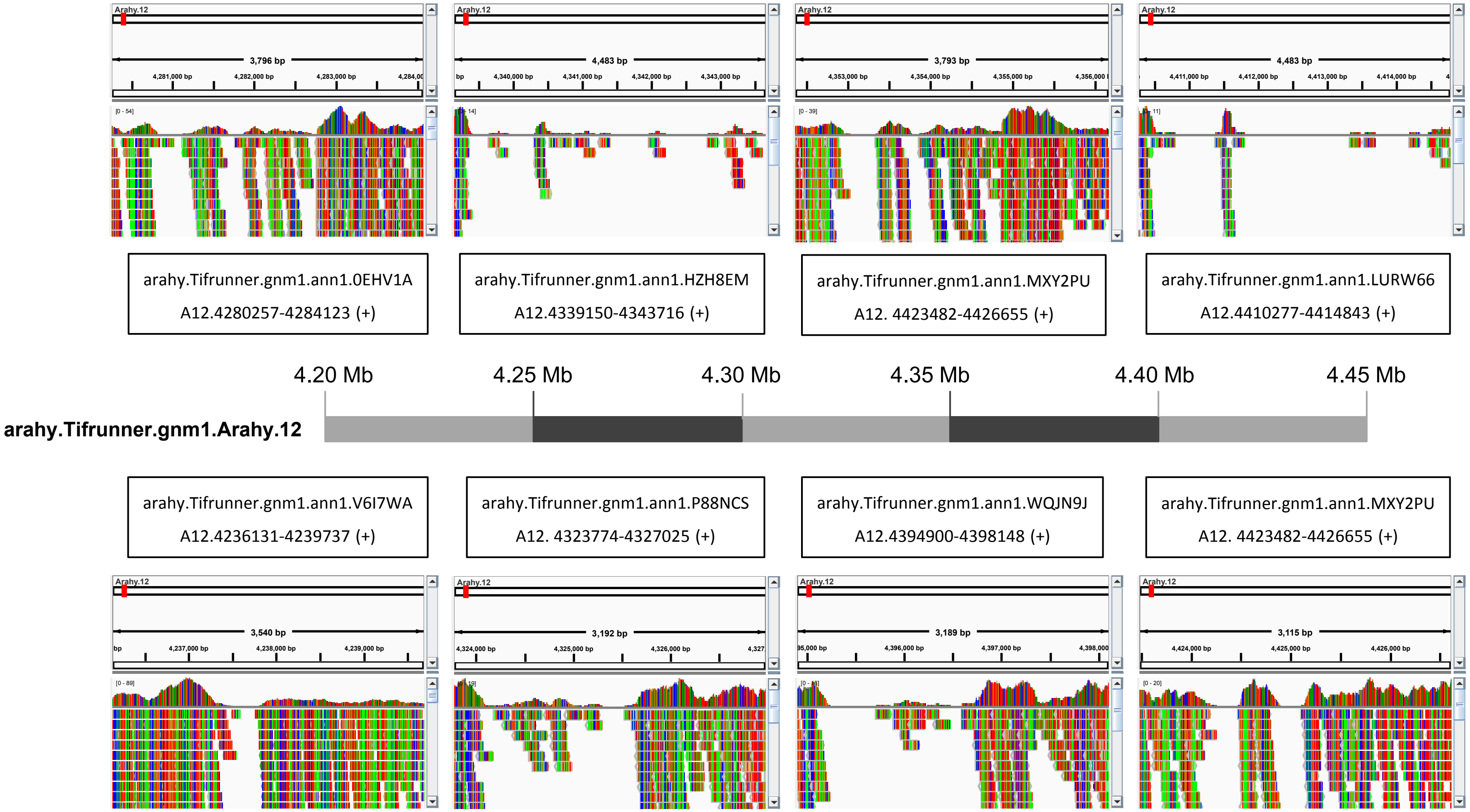

Supplementary Figure 5. Whole genome resequencing reads of Yuanza9102 aligned to the reference genome at the regions covered by eight NBS-LRR genes using IGV.
