# Supplementary material for: QTL identification, fine mapping, and marker development for breeding peanut (Arachis hypogaea L.) resistant to bacterial wilt
Source: Theor Appl Genet. 2022 Jan 20;135(4):1319–30. doi: 10.1007/s00122-022-04033-y (PMC9033696; doi:10.1007/s00122-022-04033-y)
Supplement: Supplementary file 2 — Supplementary file2 (PDF 388 KB) [file 122_2022_4033_MOESM2_ESM.pdf]

Supplementary Table S1. Descriptive statistics of the bacterial wilt resistance reaction for the two parents and the 412 RIL lines

Year[1]	YZ9102	wt09-0023	RIL Population				
	♀	♂	Range (%)	Mean (%)	SD	Kurt[2]	Skew[3]
2016_seedling	100.0%	83.3%	23.2%-100.0%	83.7%	0.2	0.5	-1.1
2016_flowering	100.0%	64.1%	13.3%-100.0%	77.0%	0.2	-0.1	-0.9
2016_pegging	100.0%	53.0%	0.0%-100.0%	72.7%	0.3	-0.5	-0.8
2016_harvesting	75.6%	35.9%	0.0%-100.0%	54.5%	0.3	-1.1	-0.3
2017_seedling	95.8%	51.8%	22.6%-100.0%	82.6%	0.2	0.5	-1.1
2017_flowering	95.8%	35.0%	0.0%-100.0%	74.4%	0.3	-0.4	-0.8
2017_pegging	85.8%	35.0%	0.0%-100.0%	69.9%	0.3	-0.8	-0.7
2017_harvesting	85.8%	20.0%	0.0%-100.0%	57.1%	0.3	-1.2	-0.3
2018_seedling	95.0%	90.0%	39.7%-100.0%	90.9%	0.1	7.9	-2.2
2018_flowering	79.0%	46.7%	3.1%-100.0%	63.2%	0.3	-1.1	-0.4
2018_pegging	79.0%	46.7%	3.1%-100.0%	60.0%	0.3	-1.2	-0.4
2018_harvesting	73.7%	0.0%	0.0%-100.0%	40.5%	0.3	-1.4	0.3

1: Year 2016, 2017: RIL 1-412; Year 2018: RIL 413-512
2: Kurtosis
3: Skewness

Supplementary Table S2. ANOVA (p-value) and the broad-sense heritability (h^2) for bacterial wilt resistance of 412 RILs at four different stages in 2016 and 2017

Effect / Trait	Seedling	Flowering	Fruiting	Harvesting
Genotype	***	***	***	***
Environment	1.0	**	**	*
G x E interaction	0.2	*	*	0.1
h^2	0.8	0.9	0.9	0.9

*, <0.05; **, <0.001; ***, <0.0001

Supplementary Table S3 Summary of the sequencing and alignment results for RILs

Sample_ID	Bases(Mb)	GC(%)	Q20(%)	Total Reads	Mapped Reads	Mapped rate
P043	16660.87	37.29	96.10	116319322	113140270	99.87%
P044	18799.43	37.24	96.13	132815093	128495282	99.85%
P260	6143.16	38.11	94.81	43054305	42962021	99.79%
P261	4397.11	38.13	94.96	30797405	30713318	99.73%
P262	5843.66	38.11	95.05	40632409	40540016	99.77%
P263	4640.84	38.17	94.94	32374414	32275385	99.69%
P264	6805.13	38.06	95.10	47499967	47367679	99.72%
P265	4536.14	38.31	95.32	31842772	31738413	99.67%
P266	4282.03	38.08	95.51	30274193	30189611	99.72%
P267	5588.60	38.32	95.61	39294699	39168930	99.68%
P268	3293.47	38.31	98.04	23075219	23047099	99.88%
P269	2212.79	38.21	98.01	15477172	15463829	99.91%
P270	4949.28	38.11	98.22	34476170	34440905	99.90%
P271	2463.11	38.31	98.10	17164146	17153342	99.94%
P272	3698.97	38.24	98.16	25753273	25731495	99.92%
P273	4953.98	37.99	98.13	34823676	34800363	99.93%
P274	4266.42	38.00	98.19	30208449	30184576	99.92%
P275	3907.32	38.32	98.18	27374749	27349534	99.91%
P276	2987.49	38.53	97.85	20867627	20847636	99.90%
P277	1875.93	38.64	97.80	13095849	13077962	99.86%
P278	4179.23	38.58	98.05	29015682	28985793	99.90%
P279	2740.44	38.69	97.95	19121689	19104842	99.91%
P280	3263.76	38.91	97.99	22710314	22687153	99.90%
P281	3624.40	38.60	97.94	25396511	25379354	99.93%
P282	3765.98	38.51	98.03	26588721	26562528	99.90%
P283	4399.29	38.68	98.01	30885056	30844210	99.87%
P284	3600.58	38.23	98.01	25259617	25228083	99.88%
P285	1634.70	38.38	97.95	11422052	11412426	99.92%
P286	3681.31	38.53	98.19	25588535	25566817	99.92%
P287	1791.51	38.13	98.07	12493782	12480525	99.89%
P288	2967.74	38.14	98.14	20670093	20653476	99.92%
P289	2493.26	38.24	98.07	17479433	17465553	99.92%
P290	3205.02	38.27	98.15	22669979	22645922	99.89%
P291	1874.77	38.74	98.14	13191757	13158129	99.75%
P292	2352.21	38.34	97.95	16425315	16402451	99.86%
P293	1626.57	38.35	97.86	11364481	11353105	99.90%
P294	3486.08	38.39	98.11	24195914	24153955	99.83%
P295	2395.56	38.36	98.01	16698334	16661027	99.78%
P296	3633.43	38.48	98.08	25560042	25539236	99.92%
P297	2801.98	38.06	97.99	19625551	19606226	99.90%
P298	2724.64	38.12	98.09	19211560	19145803	99.66%
P299	4080.83	38.06	98.08	28656433	28629394	99.91%
P301	1794.35	38.60	97.95	12532453	12515542	99.87%
P302	3977.01	38.26	98.18	27546942	27529813	99.94%
P304	3647.11	38.24	98.14	25343072	25321032	99.91%
P305	3181.93	38.55	98.05	22320123	22295774	99.89%
P306	3079.27	38.12	98.15	21682882	21665327	99.92%
P307	2713.31	38.65	98.13	19017169	18992280	99.87%
P308	3288.12	38.14	97.99	23076144	23048619	99.88%
P309	2023.75	37.85	97.93	14153988	14142008	99.92%

Sample_ID	Bases(Mb)	GC(%)	Q20(%)	Total Reads	Mapped Reads	Mapped rate
P310	4014.65	38.09	98.17	27914813	27836009	99.72%
P311	2306.98	38.24	98.06	16159179	16132853	99.84%
P312	3421.40	38.11	98.12	23937556	23918672	99.92%
P313	3641.33	37.85	98.05	25499957	25434602	99.74%
P314	3517.48	38.11	98.15	24892055	24836781	99.78%
P315	4365.48	38.16	98.12	30659907	30614539	99.85%
P316	3337.99	38.51	97.98	23401656	23379575	99.91%
P317	8837.53	37.88	95.40	62059944	61960723	99.84%
P318	2290.86	38.63	98.14	16056252	16035914	99.87%
P319	1626.10	38.43	98.06	11425411	11408539	99.85%
P320	3837.11	38.54	98.12	26719579	26691305	99.89%
P321	4146.43	38.24	98.06	29000571	28965595	99.88%
P322	3640.59	38.29	98.11	25701204	25672424	99.89%
P323	4961.96	38.33	98.12	34798739	34763240	99.90%
P324	2933.05	38.21	97.93	20522036	20501364	99.90%
P325	2018.84	38.27	97.86	14125973	14110004	99.89%
P326	2488.59	38.22	98.11	17501131	17481471	99.89%
P327	1854.13	38.10	98.01	13061562	13047963	99.90%
P328	3711.09	38.13	98.06	25962961	25935625	99.89%
P329	3418.89	38.21	98.00	24044658	24021570	99.90%
P330	2119.26	38.09	98.07	15033713	15016357	99.88%
P331	3916.37	38.31	98.07	27591982	27550583	99.85%
P332	3184.68	38.37	98.05	22280966	22258200	99.90%
P333	2066.48	38.38	97.99	14455760	14436404	99.87%
P334	4182.36	38.40	98.20	29083942	29058937	99.91%
P335	2403.25	38.62	98.12	16749297	16730632	99.89%
P336	3676.94	38.35	98.18	25583036	25565900	99.93%
P337	4097.35	38.26	98.11	28761559	28716569	99.84%
P338	4685.00	38.24	98.17	33290378	33260408	99.91%
P339	3672.79	38.89	98.18	25778671	25746598	99.88%
P341	2172.86	38.67	97.85	15178611	15144534	99.78%
P342	3763.98	38.79	98.07	26107787	26056135	99.80%
P343	2537.67	38.88	97.91	17679546	17653287	99.85%
P344	4504.09	38.49	98.01	31395069	31358540	99.88%
P345	4207.93	38.45	97.88	29514689	29463955	99.83%
P346	3518.31	38.47	98.03	24860335	24825443	99.86%
P347	4145.32	38.76	97.93	29070010	29021944	99.83%
P348	3382.38	38.31	98.04	23669148	23641548	99.88%
P349	2342.49	38.35	98.00	16377575	16355333	99.86%
P350	3454.50	38.05	98.20	23924411	23906660	99.93%
P351	2869.47	38.49	98.03	20067964	20018557	99.75%
P352	4059.69	38.08	98.11	28227196	28208835	99.93%
P353	3885.68	38.31	98.02	27263761	27226507	99.86%
P354	3346.03	37.89	98.16	23628547	23592224	99.85%
P355	3721.46	38.11	98.05	26004577	25960909	99.83%
P356	2511.85	38.24	97.96	17626926	17600647	99.85%
P357	2349.64	37.89	98.02	16514220	16502964	99.93%
P358	2877.30	37.90	98.12	20025365	20010038	99.92%
P359	3025.41	37.95	98.08	21160243	21129272	99.85%
P360	2995.37	37.90	98.04	20917241	20902455	99.93%
P361	3668.78	37.87	98.06	25794106	25769625	99.91%
P362	2284.15	37.92	98.12	16162019	16150029	99.93%

Sample_ID	Bases(Mb)	GC(%)	Q20(%)	Total Reads	Mapped Reads	Mapped rate
P363	4622.62	37.99	98.15	32535738	32492505	99.87%
P364	3751.62	38.12	97.96	26288357	26258205	99.89%
P365	2785.41	38.14	98.05	19475720	19458880	99.91%
P366	4131.53	38.24	98.12	28661522	28612305	99.83%
P367	4092.73	38.23	98.09	28552369	28528276	99.92%
P368	3893.63	38.31	98.02	27115980	27078674	99.86%
P369	4867.30	38.22	98.07	34160006	34130006	99.91%
P370	3741.81	38.29	98.07	26559875	26490110	99.74%
P371	5616.28	38.26	98.10	39487602	39450651	99.91%
P372	2675.78	38.10	98.02	18735035	18718390	99.91%
P373	2310.62	38.19	98.08	16170556	16155559	99.91%
P374	3533.75	38.20	98.17	24605918	24578968	99.89%
P375	2997.91	38.21	98.13	20915423	20897253	99.91%
P376	3061.02	38.12	98.08	21362473	21345406	99.92%
P377	3880.19	38.12	98.10	27252469	27231306	99.92%
P378	2717.44	37.92	98.14	19207872	19193205	99.92%
P380	2847.53	38.28	98.01	19865705	19609205	98.71%
P381	2788.35	38.28	98.06	19503200	19468219	99.82%
P382	3647.49	38.27	98.14	25329254	25308913	99.92%
P383	3952.35	38.33	98.13	27583015	27545283	99.86%
P384	2854.17	38.36	98.07	20014088	19990046	99.88%
P385	4446.42	38.18	98.07	31196648	31160352	99.88%
P386	3011.36	38.10	98.09	21287342	21267337	99.91%
P387	4746.78	38.33	98.14	33250372	33227135	99.93%
P388	2937.52	38.17	98.02	20657840	20638638	99.91%
P389	2333.38	38.21	98.08	16319303	16307409	99.93%
P390	2820.64	38.20	98.16	19597478	19578798	99.90%
P391	2989.65	38.26	98.16	20882492	20856721	99.88%
P393	4037.15	38.17	98.09	28392060	28343436	99.83%
P394	3068.28	38.04	98.14	21748780	21734739	99.94%
P395	4547.20	38.28	98.20	32003403	31977061	99.92%
P396	2589.12	37.27	98.07	18094608	18082601	99.93%
P397	1888.73	37.27	98.13	13214950	13205384	99.93%
P398	3300.32	37.32	98.20	22995966	22970695	99.89%
P399	2610.02	37.27	98.16	18225912	18213611	99.93%
P400	3289.25	37.26	98.13	22959319	22943420	99.93%
P401	3731.23	37.23	98.14	26179697	26160761	99.93%
P402	2400.22	37.15	98.16	16940110	16925533	99.91%
P403	4636.74	37.30	98.21	32618183	32589260	99.91%
P404	2332.69	37.42	98.05	16297323	16285346	99.93%
P405	2276.58	37.36	98.10	15924001	15910754	99.92%
P406	3281.82	37.40	98.19	22826884	22805496	99.91%
P407	2741.65	37.44	98.16	19112718	19095266	99.91%
P408	3097.17	37.34	98.12	21609862	21591565	99.92%
P409	3808.85	37.22	98.11	26772086	26754474	99.93%
P410	2842.33	37.13	98.16	20131889	20102114	99.85%
P411	4308.52	37.49	98.20	30282758	30256454	99.91%
P412	1502.20	38.99	98.06	10454183	10437794	99.84%
P413	2380.39	38.78	98.14	16665652	16651051	99.91%
P414	3371.72	38.86	98.24	23403766	23287996	99.51%
P415	2968.95	38.89	98.18	20703378	20575665	99.38%
P416	2383.50	38.74	98.12	16592992	16570615	99.87%

Sample_ID	Bases(Mb)	GC(%)	Q20(%)	Total Reads	Mapped Reads	Mapped rate
P418	2746.37	38.65	98.15	19379723	19363006	99.91%
P419	4709.26	38.78	98.22	33071439	33046615	99.92%
P420	2429.23	37.64	98.03	16983787	16962275	99.87%
P421	2537.05	37.46	98.06	17768836	17717499	99.71%
P422	3157.97	37.38	98.15	21919100	21905745	99.94%
P423	3449.44	37.43	98.13	24047080	24025013	99.91%
P424	2704.63	37.76	98.07	18830699	18815115	99.92%
P425	4558.41	37.38	98.08	32078794	32059713	99.94%
P426	1756.38	37.33	98.04	12321008	12312438	99.93%
P427	4939.96	37.48	98.15	34676812	34530526	99.58%
P428	3127.68	37.59	98.06	21889153	21869643	99.91%
P429	3110.31	37.62	98.12	21777837	21761996	99.93%
P430	3014.45	37.58	98.18	20846115	20829413	99.92%
P431	3958.67	37.75	98.17	27556207	27527570	99.90%
P432	3504.01	37.58	98.12	24389594	24371553	99.93%
P433	5520.01	37.67	98.13	38793765	38763258	99.92%
P434	2852.98	37.47	98.09	20185972	20135631	99.75%
P435	4484.29	37.82	98.20	31507275	31479753	99.91%
P437	2621.20	37.55	98.02	18307956	18298142	99.95%
P438	2829.69	37.88	98.10	19578183	19562884	99.92%
P439	4086.50	37.48	98.07	28482242	28423250	99.79%
P440	3162.45	37.64	98.03	21992440	21943314	99.78%
P441	4711.62	37.45	98.03	33090444	33045878	99.87%
P442	3160.63	37.39	98.03	22366371	22348461	99.92%
P443	6334.68	37.65	98.13	44649767	44615503	99.92%
P444	2227.89	38.72	98.01	15519243	15503171	99.90%
P445	1954.94	38.90	98.08	13629693	13613714	99.88%
P446	2933.93	38.69	98.14	20301902	20287301	99.93%
P447	3330.08	38.78	98.09	23227140	23206865	99.91%
P448	2824.46	38.86	98.07	19632546	19611898	99.89%
P449	3513.82	38.78	98.08	24610635	24589587	99.91%
P450	2895.95	38.49	98.04	20425167	20401721	99.89%
P451	3918.58	38.66	98.13	27405751	27379396	99.90%
P452	3300.85	37.38	97.95	23085416	23070853	99.94%
P453	2075.41	37.39	97.90	14536069	14522825	99.91%
P454	5097.55	37.40	98.09	35379543	35360441	99.95%
P455	2476.28	37.48	97.96	17247837	17228982	99.89%
P456	3692.80	37.35	98.02	25704057	25685301	99.93%
P457	3324.68	37.35	97.94	23343110	23302788	99.83%
P458	2763.10	37.27	98.03	19515222	19498547	99.91%
P459	4006.49	37.45	98.04	28151321	28124509	99.90%
P460	3668.39	37.97	98.04	25649939	25620292	99.88%
P461	3068.87	37.90	98.03	21473686	21456308	99.92%
P462	4498.12	37.79	98.14	31279915	31257829	99.93%
P464	4264.54	37.72	98.09	29620947	29596720	99.92%
P465	4721.84	37.80	98.02	33210361	33185851	99.93%
P466	3476.60	37.83	98.05	24587969	24546479	99.83%
P467	3657.23	38.47	98.08	25694999	25622278	99.72%
P468	3357.79	38.60	98.15	23509904	23495848	99.94%
P469	1841.34	38.66	98.07	12877063	12868438	99.93%
P470	3986.44	39.06	98.23	27734609	27697476	99.87%
P471	2298.55	38.68	98.10	16009882	15994827	99.91%

Sample_ID	Bases(Mb)	GC(%)	Q20(%)	Total Reads	Mapped Reads	Mapped rate
P472	4116.42	38.70	98.20	28735448	28691876	99.85%
P473	3887.05	38.64	98.08	27389830	27368660	99.92%
P474	3072.50	38.68	98.15	21710913	21686876	99.89%
P475	3819.75	38.68	98.13	26782826	26761967	99.92%
P476	2926.50	37.34	98.05	20438603	20424905	99.93%
P477	2056.21	37.34	98.02	14363061	14351303	99.92%
P478	2816.84	37.39	98.13	19519625	19498585	99.89%
P479	2539.36	37.38	98.04	17684199	17669901	99.92%
P480	3615.15	37.34	98.09	25208520	25192187	99.94%
P481	3371.23	37.31	98.02	23690828	23671023	99.92%
P482	3620.99	37.15	98.02	25690158	25668992	99.92%
P483	4393.91	37.36	98.08	30866520	30837773	99.91%
P484	6350.95	36.93	97.56	44617634	44574395	99.90%
P485	3437.67	36.94	97.47	24012832	23958190	99.77%
P486	5455.76	37.03	97.54	38025539	37966506	99.84%
P487	3673.54	36.95	97.43	25639922	25609031	99.88%
P488	5987.83	36.91	97.57	41842420	41807604	99.92%
P489	3549.01	37.01	97.50	24899130	24868066	99.88%
P490	3337.05	36.99	97.56	23618992	23592325	99.89%
P491	3568.07	37.50	97.55	25019752	24972190	99.81%
P492	6316.63	37.01	97.59	44337637	44301743	99.92%
P493	4763.50	36.59	97.49	33359129	33329521	99.91%
P494	6142.13	36.55	97.57	42699606	42670846	99.93%
P495	4541.81	36.57	97.48	31631385	31601928	99.91%
P496	4976.51	36.67	97.59	34716388	34677670	99.89%
P497	5377.54	36.98	97.53	37893643	37849306	99.88%
P498	3809.68	36.57	97.59	26948991	26920380	99.89%
P499	6373.57	36.90	97.58	44911789	44867110	99.90%
P500	7421.50	36.60	96.74	43849983	43770358	99.82%
P502	3255.37	36.49	96.75	22828318	22781374	99.79%
P503	1753.47	36.50	96.65	12312817	12282593	99.75%
P504	6868.86	36.30	96.74	48092264	47953762	99.71%
P505	3736.05	36.41	96.68	26170149	26119956	99.81%
P506	3450.54	36.37	96.71	24413636	24356140	99.76%
P507	3954.16	37.10	96.79	27697092	27645351	99.81%
P508	4556.32	37.20	96.64	31936528	31864308	99.77%
P509	3420.46	37.10	96.47	23913429	23854549	99.75%
P511	3150.23	37.46	96.49	22045666	21980562	99.70%
P512	3075.75	37.19	96.66	21333312	21282544	99.76%
P513	3116.36	37.32	96.52	21840588	21787365	99.76%
P514	3661.05	37.29	96.55	25959099	25881245	99.70%
P515	3073.83	37.96	96.63	21547905	21496562	99.76%
P516	5040.17	38.08	97.68	35172817	35144511	99.92%
P517	5603.62	36.95	96.62	39187372	39097757	99.77%
P518	3482.15	36.91	96.46	24308215	24247340	99.75%
P519	5339.86	36.93	96.62	37113774	37022386	99.75%
P520	3824.45	36.89	96.48	26644093	26582182	99.77%
P521	6810.45	36.69	96.64	47726648	47629139	99.80%
P522	3554.21	37.52	96.54	25000388	24935562	99.74%
P523	3288.96	36.88	96.61	23276421	23231129	99.81%
P524	4397.39	37.50	96.66	30948692	30856800	99.70%
P525	5843.36	37.09	96.29	40963929	40814626	99.64%

Sample_ID	Bases(Mb)	GC(%)	Q20(%)	Total Reads	Mapped Reads	Mapped rate
P526	3680.64	36.94	96.12	25686646	25574720	99.56%
P527	4815.82	37.19	96.22	33362209	33241224	99.64%
P528	4154.27	37.25	96.11	28953002	28815301	99.52%
P529	5803.84	37.00	96.29	40422800	40275114	99.63%
P530	6376.65	36.91	96.19	44844093	44667830	99.61%
P531	3495.07	37.13	96.21	24672673	24580150	99.63%
P532	3962.87	37.46	96.28	27789818	27607155	99.34%
P533	7197.81	36.64	96.06	50617921	50479155	99.73%
P534	3805.56	36.85	96.00	26649091	26566511	99.69%
P535	4062.56	36.90	96.14	28205458	28130077	99.73%
P536	3376.54	37.08	95.93	23549169	23463185	99.63%
P537	4322.75	36.91	96.07	30053220	29960458	99.69%
P538	4154.09	36.82	96.13	29194745	29088630	99.64%
P539	2170.33	36.90	96.24	15299856	15262305	99.75%
P540	3270.65	37.28	96.15	23012811	22944921	99.70%
P541	3968.07	37.15	98.28	27886225	27872658	99.95%
P542	2323.56	37.25	98.24	16256811	16247575	99.94%
P543	2940.95	37.12	98.39	20429186	20414883	99.93%
P544	2656.67	37.10	98.31	18578254	18567669	99.94%
P545	3903.86	37.11	98.36	27247231	27232484	99.95%
P546	3651.53	37.13	98.32	25687354	25674221	99.95%
P547	2586.27	37.03	98.46	18303814	18294233	99.95%
P548	3594.61	37.14	98.45	25257420	25244255	99.95%
P549	1796.65	37.28	98.45	12569677	12563271	99.95%
P550	4318.58	38.09	97.68	30156621	30137726	99.94%
P551	1957.83	37.20	98.49	13781666	13773546	99.94%
P552	2067.62	37.10	98.56	14378716	14371408	99.95%
P553	2336.86	37.33	98.55	16344839	16334487	99.94%
P554	2583.33	37.29	98.53	18046685	18030988	99.91%
P555	3710.75	37.22	98.56	26192290	26177078	99.94%
P556	2557.84	37.26	98.70	18228935	18218873	99.94%
P557	3941.42	37.24	98.71	27718385	27694902	99.92%
P558	2709.91	37.30	98.27	18923327	18882300	99.78%
P559	3225.89	37.35	98.31	22548576	22535769	99.94%
P560	3694.55	37.20	98.40	25638247	25620563	99.93%
P561	3701.99	37.39	98.38	25751852	25728585	99.91%
P563	5062.85	37.26	98.41	35555682	35531624	99.93%
P564	4359.48	37.14	98.51	30873896	30850300	99.92%
P565	6279.55	37.31	98.54	44080391	44054420	99.94%
P566	3111.12	37.14	98.24	21778499	21766109	99.94%
P567	2663.31	37.21	98.27	18592034	18577787	99.92%
P568	3008.03	37.04	98.35	20855498	20843994	99.94%
P569	3726.35	37.14	98.35	25994130	25954731	99.85%
P570	3216.12	37.15	98.32	22332734	22319243	99.94%
P571	5243.39	37.12	98.38	36843103	36825406	99.95%
P572	3842.02	36.93	98.48	27177088	27159274	99.93%
P573	6643.39	37.12	98.52	46811526	46776064	99.92%
P574	2557.98	37.73	98.23	17854997	17837600	99.90%
P575	2460.50	37.62	98.28	17179768	17164397	99.91%
P576	3289.19	37.59	98.36	22860423	22838852	99.91%
P577	3918.32	37.96	98.36	27377942	27336530	99.85%
P578	3143.45	37.73	98.31	21886373	21855532	99.86%

Sample_ID	Bases(Mb)	GC(%)	Q20(%)	Total Reads	Mapped Reads	Mapped rate
P579	5080.48	37.62	98.36	35719179	35651120	99.81%
P580	3136.51	37.54	98.42	22158216	22120554	99.83%
P581	5248.55	37.83	98.50	36751666	36725526	99.93%
P582	2795.66	37.19	98.19	19553784	19544480	99.95%
P583	2517.97	37.26	98.25	17660030	17639082	99.88%
P584	2547.55	37.19	98.31	17693431	17677074	99.91%
P585	4058.58	37.21	98.31	28400842	28390416	99.96%
P586	3371.83	37.25	98.28	23531327	23518346	99.94%
P587	3761.81	37.13	98.33	26461241	26440096	99.92%
P588	6132.17	37.30	98.47	20556516	20516059	99.80%
P589	6132.17	37.30	98.47	43164469	43129293	99.92%
P590	2658.90	37.52	98.26	18457230	18421017	99.80%
P591	5057.25	37.25	98.30	35492086	35471983	99.94%
P592	3519.68	37.12	98.40	24530085	24500834	99.88%
P593	4250.25	37.27	98.37	29738384	29721858	99.94%
P594	3608.40	37.25	98.35	25206823	25188518	99.93%
P595	6143.64	37.07	98.40	43261085	43237453	99.95%
P596	4301.30	37.14	98.49	30476944	30453318	99.92%
P597	4614.63	37.29	98.53	32370164	32336512	99.90%
P598	3059.89	37.67	98.33	21411751	21390076	99.90%
P599	3358.19	37.56	98.37	23565744	23552661	99.94%
P600	3142.65	37.41	98.45	21810048	21800880	99.96%
P601	3200.83	37.56	98.43	22295472	22271415	99.89%
P602	3946.92	37.50	98.42	27563544	27549761	99.95%
P603	4212.84	37.67	98.46	29553698	29484830	99.77%
P604	3395.26	37.48	98.55	24030693	24017850	99.95%
P605	5464.40	37.53	98.59	38332226	38312970	99.95%
P607	3084.02	38.40	97.86	21554108	21531402	99.89%
P608	3579.33	38.37	97.97	24864835	24846348	99.93%
P609	4043.74	38.44	97.97	28170065	28146377	99.92%
P610	3020.65	38.38	97.90	21005373	20988032	99.92%
P611	5125.00	38.45	97.96	35975115	35932714	99.88%
P612	3919.55	38.33	98.05	27684906	27659412	99.91%
P613	5189.59	38.49	98.12	36257819	36231847	99.93%
P614	2295.02	37.15	97.65	16049900	16037728	99.92%
P615	2796.54	37.03	97.70	19626996	19613069	99.93%
P616	3212.26	37.09	97.81	22348689	22274191	99.67%
P617	3994.14	37.23	97.80	27951294	27921235	99.89%
P618	2909.65	37.24	97.71	20301569	20285993	99.92%
P620	2835.18	36.96	97.68	20098826	20083517	99.92%
P621	4915.92	37.24	97.81	34547884	34520823	99.92%
P622	2423.49	37.27	98.03	16971255	16951313	99.88%
P623	3041.89	37.20	98.09	21344549	21327917	99.92%
P624	3954.95	38.13	97.66	27415206	27359585	99.80%
P625	3025.01	37.17	98.17	21021160	20994038	99.87%
P626	3759.17	37.25	98.14	26263283	26241024	99.92%
P627	2147.93	37.21	98.10	14992001	14980735	99.92%
P628	4810.84	37.22	98.12	33826374	33800756	99.92%
P629	2899.43	37.07	98.09	20489182	20471645	99.91%
P630	6117.01	37.25	98.19	43006224	42960022	99.89%
P631	2876.35	37.12	97.77	20063763	20047144	99.92%
P632	4536.40	37.15	97.86	31898447	31689647	99.35%

Sample_ID	Bases(Mb)	GC(%)	Q20(%)	Total Reads	Mapped Reads	Mapped rate
P633	4308.74	37.16	97.93	29963378	29924261	99.87%
P634	4845.12	37.10	97.88	33861677	33829103	99.90%
P635	3805.46	37.16	97.84	26552626	26527243	99.90%
P636	5841.53	36.98	97.89	41126928	41074252	99.87%
P637	4167.53	37.04	97.88	29632592	29608569	99.92%
P639	3817.03	37.05	97.72	26761076	26742930	99.93%
P640	3295.27	37.01	97.80	23095600	23079214	99.93%
P641	3818.75	37.04	97.86	26547355	26527010	99.92%
P642	3877.84	37.02	97.82	27009032	26981365	99.90%
P643	3433.74	37.00	97.77	23885716	23868136	99.93%
P644	4592.40	36.99	97.80	32233925	32207657	99.92%
P645	3234.80	37.47	97.77	22938557	22893798	99.80%
P646	4358.38	37.04	97.85	30738975	30716179	99.93%
P647	2405.12	37.14	97.89	16849342	16831650	99.90%
P648	2308.16	38.47	95.97	15910801	15825733	99.47%
P649	2427.61	37.13	98.09	16898323	16878016	99.88%
P650	2427.89	37.08	97.95	16988201	16905945	99.52%
P651	3090.19	37.06	97.99	21589402	21550640	99.82%
P652	1924.49	37.20	97.90	13487989	13478566	99.93%
P653	1820.18	37.06	98.05	12934886	12926930	99.94%
P654	2267.88	37.23	98.01	15852787	15839025	99.91%
P655	2761.80	38.06	97.93	19279047	19260567	99.90%
P656	2042.15	38.22	97.92	14258946	14218402	99.72%
P657	3144.76	38.14	98.07	21852137	21834116	99.92%
P658	2589.20	38.08	97.95	18035149	17981304	99.70%
P659	3435.09	38.07	97.99	23904474	23884619	99.92%
P660	3967.03	37.94	97.95	27867556	27847734	99.93%
P661	2183.23	38.06	97.99	15396911	15380140	99.89%
P662	2922.76	38.10	97.99	20465225	20445704	99.90%
P663	2919.21	38.05	98.04	20344644	20330652	99.93%
P664	2228.49	38.32	98.01	15576464	15563807	99.92%
P665	2573.85	38.17	98.16	17795315	17777480	99.90%
P666	2296.93	38.08	98.05	15934558	15920739	99.91%
P667	3725.51	38.02	98.09	25949255	25926102	99.91%
P668	4099.52	38.07	97.67	28524199	28493159	99.89%
P669	3323.13	38.01	98.03	23316705	23297003	99.92%
P671	3688.42	38.10	98.10	25853412	25795178	99.77%
P672	3736.85	37.94	97.81	26110657	26083169	99.89%
P673	2879.66	38.00	97.78	20115048	20103710	99.94%
P674	3582.54	38.07	97.94	24798575	24778157	99.92%
P675	3091.05	38.15	97.80	21491510	21451991	99.82%
P676	3833.61	37.94	97.87	26576019	26553530	99.92%
P677	4398.58	37.94	97.79	30831412	30716825	99.63%
P678	2349.42	37.87	97.80	16504338	16489853	99.91%
P679	4510.76	38.01	97.82	31616252	31588189	99.91%
P680	4269.53	36.87	97.81	29927607	29907626	99.93%
P681	2922.23	36.98	97.81	20459408	20440020	99.91%
P682	2376.97	36.87	97.96	16625733	16612224	99.92%
P683	2883.20	37.32	97.85	20141901	20125863	99.92%
P684	3310.42	36.86	97.88	23033878	23019603	99.94%
P685	4507.76	36.80	97.82	31674962	31637196	99.88%
P686	2678.41	36.68	97.88	18835409	18807096	99.85%

Sample_ID	Bases(Mb)	GC(%)	Q20(%)	Total Reads	Mapped Reads	Mapped rate
P687	3828.69	37.38	97.88	26830287	26800159	99.89%
P688	3970.59	37.53	97.45	27759320	27730096	99.89%
P689	2141.14	37.67	97.42	14890635	14875116	99.90%
P690	5269.84	37.52	97.31	36584589	36557236	99.93%
P691	3478.10	37.66	97.32	24174912	24147511	99.89%
P692	3341.92	37.61	97.38	23124859	23067273	99.75%
P693	4907.05	37.54	97.41	34381498	34338415	99.87%
P694	3462.64	37.40	97.51	24377517	24353124	99.90%
P695	2896.27	38.01	97.52	20134522	20103770	99.85%
P696	3591.21	37.25	97.63	25046304	25025390	99.92%
P697	2558.68	37.23	97.62	17855085	17813523	99.77%
P698	5097.61	37.66	97.50	35876839	35839387	99.90%
P699	2823.41	37.25	97.53	19799961	19777961	99.89%
P700	4697.61	37.22	97.57	32705668	32653550	99.84%
P701	3702.90	37.06	97.59	26074918	25557536	98.02%
P702	4035.15	37.01	97.69	28540068	28502605	99.87%
P703	1702.04	38.02	97.68	11779592	11730856	99.59%
P704	2339.67	37.57	96.88	16319554	16290249	99.82%
P705	1911.22	37.67	96.82	13333667	13317238	99.88%
P706	3148.71	37.54	96.66	21770570	21728242	99.81%
P707	2702.77	37.83	96.69	18819781	18795119	99.87%
P709	3576.87	37.48	96.79	25087464	25048258	99.84%
P710	2572.78	37.46	96.90	18124228	18104259	99.89%
P711	3121.92	37.60	96.91	21855717	21824308	99.86%
P712	2262.21	38.79	97.03	15752294	15722199	99.81%
P713	5935.08	38.12	95.46	41105657	41006894	99.76%
P714	1900.40	38.41	96.87	13143427	13066227	99.41%
P715	3036.87	38.52	96.89	21125217	21102803	99.89%
P716	3136.43	38.51	96.95	21753300	21730369	99.89%
P717	3360.15	38.51	96.97	23483481	23448488	99.85%
P718	2045.68	38.26	97.07	14507322	14486474	99.86%
P719	3185.71	38.47	97.08	22241335	22214632	99.88%
P720	2546.80	37.80	97.53	17861527	17839947	99.88%
P721	2238.83	38.02	97.52	15581371	15566059	99.90%
P722	4642.67	37.76	97.38	32150784	32122741	99.91%
P723	3987.40	37.86	97.42	27730741	27698124	99.88%
P724	4223.34	37.79	97.49	29333770	29188631	99.51%
P725	3652.46	37.85	97.50	25749141	25726411	99.91%
P726	2772.10	37.67	97.56	19653477	19634917	99.91%
P727	4087.57	37.83	97.59	28538522	28505127	99.88%
P728	2421.61	37.70	96.89	16998499	16929980	99.60%
P729	2254.44	37.70	96.86	15751919	15629664	99.22%
P730	3471.33	37.81	96.75	24193387	24161275	99.87%
P731	3103.83	37.72	96.73	21583460	21560792	99.89%
P732	3286.31	37.73	96.82	22968433	22939576	99.87%
P733	4526.81	37.73	96.88	31769305	31719305	99.84%
P734	3144.24	37.59	97.03	22189860	22167290	99.90%
P735	4048.79	37.60	97.00	28380555	28347075	99.88%
P736	3055.04	37.70	97.37	21362408	21344717	99.92%
P737	1591.59	37.75	97.38	11062346	11028614	99.70%
P738	3675.11	37.56	97.23	25423193	25401452	99.91%
P739	1954.16	37.76	97.23	13545045	13504194	99.70%

Sample_ID	Bases(Mb)	GC(%)	Q20(%)	Total Reads	Mapped Reads	Mapped rate
P740	5011.06	37.80	97.31	34967759	34942485	99.93%
P741	3837.78	37.96	97.33	26894421	26847325	99.82%
P742	3808.31	37.51	97.42	26994999	26956407	99.86%
P743	4212.51	37.63	97.41	29671471	29648148	99.92%
P744	1707.93	38.03	97.43	11930456	11910824	99.84%
P745	6839.06	37.78	95.53	47574611	47460405	99.76%
P746	3692.60	37.38	97.34	25650134	25620533	99.88%
P747	2551.37	37.64	97.45	17727391	17694233	99.81%
P748	2421.09	37.44	97.38	16799063	16787205	99.93%
P749	3465.12	37.33	97.56	24344964	24313022	99.87%
P750	2213.27	37.41	97.46	15621208	15594537	99.83%
P751	4354.42	37.51	97.62	30600005	30576803	99.92%
P752	2617.05	37.65	97.30	18234346	18218257	99.91%
P753	2319.10	37.68	97.45	16193618	16168617	99.85%
P754	3679.56	37.63	97.20	25395473	25369065	99.90%
P755	3658.97	37.93	97.31	25454034	25419999	99.87%
P756	3345.14	37.69	97.25	23288354	23229360	99.75%
P757	4256.45	37.82	97.43	29907467	29853947	99.82%
P758	3295.12	37.46	97.42	23220655	23197550	99.90%
P759	4992.05	37.68	97.50	34965560	34925471	99.89%
P760	4223.15	37.83	96.63	29422268	29360834	99.79%
P762	4309.54	37.78	96.82	29825484	29693702	99.56%
P763	3861.57	37.83	96.72	26776493	26716800	99.78%
P764	4874.72	37.77	96.70	33909510	33846859	99.82%
P765	5068.13	38.05	96.87	35556440	35481067	99.79%
P766	3668.38	38.19	96.87	25889139	25826709	99.76%
P767	3488.97	37.92	96.96	24314914	24275676	99.84%
P768	4348.00	36.94	95.54	30375861	30327109	99.84%
P769	4808.65	37.04	95.76	33794552	33751585	99.87%
P770	4617.64	37.03	95.72	32054082	32015795	99.88%
P771	4148.74	36.96	95.66	28850638	28785945	99.78%
P772	4968.86	37.00	95.59	34671474	34623568	99.86%
P773	5082.79	37.04	95.80	35721263	35665068	99.84%
P774	3860.40	36.83	95.65	27293465	27255941	99.86%
P775	5431.76	37.07	95.87	38150269	38104083	99.88%
P776	3345.89	38.41	96.48	23291740	23204757	99.63%
P777	3141.14	38.24	96.62	21881537	21837287	99.80%
P778	4499.25	38.30	96.57	31230556	31163237	99.78%
P779	4193.25	38.29	96.55	29150656	29099982	99.83%
P780	3915.54	38.17	96.49	27181673	26887890	98.92%
P781	4841.53	38.16	96.70	33925861	33878448	99.86%
P782	3453.38	38.28	96.58	24357767	24319498	99.84%
P783	5187.13	38.39	96.73	36456071	36382682	99.80%
P784	4261.08	37.18	95.81	29830207	29776736	99.82%
P785	4092.56	37.20	95.97	28624282	28577177	99.84%
P786	4416.22	37.29	96.01	30658418	30540204	99.61%
P787	4481.51	37.19	95.96	31203988	31149548	99.83%
P788	4425.82	37.08	95.87	30871398	30812414	99.81%
P789	5573.71	37.05	96.09	39207042	39115033	99.77%
P790	3862.97	37.18	96.11	27325573	27290993	99.87%
P791	4670.40	37.32	96.26	32815308	32753816	99.81%
P792	4648.21	37.54	95.39	32461865	32326430	99.58%

Sample_ID	Bases(Mb)	GC(%)	Q20(%)	Total Reads	Mapped Reads	Mapped rate
P793	3537.52	37.68	95.54	24673452	24560737	99.54%
P794	4848.37	37.58	95.50	33625441	33545181	99.76%
P795	6959.79	38.11	97.80	48356424	48313876	99.91%
P796	5639.80	37.70	95.50	39398399	39316433	99.79%
P797	5404.54	37.63	95.42	37695441	37586018	99.71%
Total	1918802.63	-	-	13399642792	13372048129	-

Note: P043 is the female parent (Yuanza9102) and P044 is the male parent (wt09-0023).

Supplementary Table S4 Chromosomal locations and primer sequences for the KASP markers used in this study

ID	Chrom	Position	Primer_AlleleFAM	Primer_AlleleHEX	Primer_Common	AlleleFAM	AlleleHEX
A12.3337247	Arahy.12	3337247	CGTACACCTTATTAATTTAGAGGGGG	ACGTACACCTTATTAATTTAGAGGGGA	GCACTGGCGGAGCCACGTTATA	G	A
A12.3365381	Arahy.12	3365381	TTTTAACCTAAAGTACTATGGGTATCACT	AACCTAAAGTACTATGGGTATCACG	ATCAAATACATACTTAGTGGTGGCTGATTT	A	C
A12.3428172	Arahy.12	3428172	GTAATTTGGTCATGACTAGTGTTAGAAAAT	GTAATTTGGTCATGACTAGTGTTAGAAAAA	GATGTTAAGAATCTGTTGTTTTTTAGTATA	A	T
A12.4058716	Arahy.12	4058716	CAACCTGTGTGCATGACCTCAC	CCAACCTGTGTGCATGACCTCAT	CGCCCTCACTAAGCTTACTATGGAT	C	T
A12.4079137	Arahy.12	4079137	AGTTGGGCCGGTTCCAGGC	CTAGTTGGGCCGGTTCCAGGA	TCCGAACCGAAGAAACCCTCTAGAA	C	A
A12.4097252	Arahy.12	4097252	CAAAACAGCAAGTTGAAGAATGTTAG	CAAAACAGCAAGTTGAAGAATGTTAC	TACAATCCTTTTAGTGGCCTTACTC	G	C
A12.4237459	Arahy.12	4237459	CATAATCAACTCAATAAGCAAGTAC	CAACATAATCAACTCAAAAACCTCAAAT	CAGATTGAGTTACAATCACTTGCCAC	C	T
A12.4240574	Arahy.12	4240574	GAGAAAATAGGGTTATTTTCTGCCTTGTT	GAAAATAGGGTTATTTTCTGCCTTGTC	TACAGTAAGATGTCAGAACTTGAGCCTT	A	G
A12.4255141	Arahy.12	4255141	AAAAGCAGTCTCAAAATTCATAGTTAGTC	CAAAAGCAGTCTCAAAATTCATAGTTAGTT	ATAAAGTATAGTCCTCTAAGATAACATAAA	G	A
A12.4255592	Arahy.12	4255592	ATAATGATAACATAACAGATGTCAATAAATG	CTATAATGATAACATAACAGATGTCAATAAATA	CATGATGGTATAAATGGTTATGACACGTAT	C	T
A12.4277067	Arahy.12	4277067	TGGCTTTCAGTGGAAGCTTCAAGA	GGCTTTCAGTGGAAGCTTCAAGG	CCCACCTTCAAATACTTGAACCCTAAATT	A	G
A12.4278514	Arahy.12	4278514	GAGCTTGACACTGCGGTCGC	AGAGCTTGACACTGCGGTCGT	AGCTCCGATCGCAGCACCGTAT	C	T
A12.4471816	Arahy.12	4471816	AAAAATTTAATTAAGTGGCATTTCATTTAAGAA	AAAAATTTAATTAAGTGGCATTTCATTTAAGAG	GAGATAATTGTGAAGGATCGGATGAGATA	A	G
A12.4574468	Arahy.12	4574468	ATTATACTTTAATATATATTCACTACTTATACA	ATACTTTAATATATATTCACTACTTATACG	TCCTTCCTAGCTAGCTTGTTTCTTTTAAAA	A	G
A12.4628249	Arahy.12	4628249	GAACTTATCTGCCTACGTACCGAA	AACTTATCTGCCTACGTACCGAG	GGCTTCATGCCAGCTGTCCGAT	T	C
A12.4875700	Arahy.12	4875700	GTCCGATTTCTGTACCTCAAATTTTTAAT	GTCCGATTTCTGTACCTCAAATTTTTAAA	ACAAATCGAACCGTGCGATTTGTGTTAAA	A	T
A12.5049380	Arahy.12	5049380	TTTGCATTGTGTATACAACAACTCATTAG	AAATTTTGCATTGTGTATACAACAACTCATTAA	GACGAACTAATGACTAATTCGTTGTATATT	C	T

Supplementary Table S5 Bacterial wilt resistance of advanced generation breeding materials

ID	Accession	Pedigree	Derived from Yuanza9102	Survival Rate %
1	YZ9102	baisha1016/A.chacoense	Yes	82.40%
2	2015-W196	(baisha1016/(fuqing/A.chacoense))/(baitu131/YZ9102)-0-0-Sd-12-2-1-1	Yes	93.20%
3	2015-W215	YZ9102/02CG005-N-0-Sd-Sd-Sd-Sd-2	Yes	96.70%
4	2015-W219	YZ9102/((baisha1016/A.vill)/luhua9)-N-0-7-2-2-2	Yes	98.10%
5	2015-W221	YZ9102/((baisha1016/A.vill)/luhua9)-N-0-29-1-2-2	Yes	93.80%
6	2015-W223	YZ9102/((baisha1016/A.vill)/luhua9)-N-0-3-1-1-1-2	Yes	89.10%
7	2015-W227	YZ9102/(yuhua7/(fuqing/(lankaoduoli/A.chacoense)))-N-0-Sd-2-2-1	Yes	76.60%
8	2015-W233	YZ9102/(yuhua7/(fuqing/(lankaoduoli/A.chacoense)))-N-0-Sd-Sd-Sd-5-1	Yes	75.40%
9	2015-W244	YZ9102/(yuhua11/((lankaoduoli/A.chacoense)/(xu7506-57/P12)))-N-0-Sd-Sd-Sd-112(N)-1-1	Yes	85.30%
10	2015-W312	(YZ9102/shiyouhong4)/(mH8/baipi)-N-0-Sd-1-3-1	Yes	96.70%
11	2015-W361	YZ9102/wt08-0932(w1004)-N1-0-41-2-2	Yes	96.70%
12	2015-W362	YZ9102/wt08-0932(w1004)-N1-0-70-1-1	Yes	96.30%
13	2015-W363	YZ9102/wt09-0023(w1005)-N2-0-4-2-1	Yes	96.20%
14	2015-W365	YZ9102/wt09-0023(w1005)-N1-0-Sd-270(N)-Sd-1	Yes	97.30%
15	2015-W384	(YZ9102/shitouqi)/wt08-0932-N-0-3-1-1	Yes	74.40%
16	2017-W108	YZ9102/((baisha1016/A.vill)/luhua9)-N-0-28-1-1-1-1-1	Yes	76.60%
17	2017-W1107	YZ9102/02CG005-N-0-82-1-1-1-2-2	Yes	76.00%
18	2017-W115	YZ9102/(yuanza9307/((baisha1016/A.villosa)/luhua9))-0-0-8-1-2-1	Yes	95.40%
19	2017-W1204	YZ9102/(yuanza9307/((baisha1016/A.villosa)/luhua9)))-0-0-34-1-1	Yes	81.50%
20	2017-W1205	YZ9102/(yuanza9307/((baisha1016/A.villosa)/luhua9))-0-0-68-1-3	Yes	83.70%
21	2017-W1511	YZ9102/DF12-0-3-1-7(N)-3	Yes	83.20%
22	2017-W197	(YZ9102/(baisha1016/(yuhua7/A.villosa)))/YuhuaY09-1-0-0-Sd-sd-sd-1	Yes	92.00%
23	2017-W211	(YZ9102/shitouqi)/wt09-0023-N-0-Sd-sd-sd-9	Yes	96.40%
24	2017-W235	YZ9102/(yuanza9307/((baisha1016/A.villosa)/luhua9))-0-0-sd-sd-16	Yes	76.50%
25	2017-W236	YZ9102/(yuanza9307/((baisha1016/A.villosa)/luhua9))-0-0-sd-sd-22	Yes	100.00%
26	2017-W239	YZ9102/(yuanza9307/((baisha1016/A.villosa)/luhua9))-0-0-sd-sd-52	Yes	72.40%
27	2017-W241	YZ9102/(yuanza9307/((baisha1016/A.villosa)/luhua9))-0-0-sd-sd-56	Yes	85.30%
28	2017-W322	yuhua15/((YZ9102/yuhua11)/((baisha1016/A.vill)/luhua9))-0-0-sd-4	Yes	75.60%
29	2017-W328	YZ9102/w9908-N-0-Sd-Sd-Sd-10-Sd-1	Yes	96.50%
30	2017-W329	YZ9102/w9908-N-0-Sd-Sd-Sd-12-Sd-1	Yes	94.40%
31	2017-W332	YZ9102/w9908-N-0-Sd-Sd-Sd-42-Sd-2	Yes	82.20%
32	shanghua5	yuhua9414/YZ9102	Yes	88.90%
33	yuhua23	zheng9316/YZ9102	Yes	84.70%
34	yuanza6	YZ9102/shitouqi	Yes	86.20%
35	yueyou18	yueyou13/YZ9102	Yes	81.30%
36	2015-W217	YZ9102/(yuhua4/zheng8903)-N-0-Sd-Sd-Sd-Sd-3	Yes	64.90%
37	2015-W303	(YZ9102/yuhua11)/((baisha1016/A.villosa)/luhua9)-N-0-Sd-4-1-2-1	Yes	60.60%
38	2017-W1104	tianfu15/(baitu131/YZ9102)-0-0-Sd-Sd-Sd-2-1-1	Yes	0.00%
39	2017-W1303	yuhua15/((YZ9102/yuhua11)/((baisha1016/A.villosa)/luhua9))-0-0-1	Yes	2.60%
40	2017-W147	yuhua15/((YZ9102/yuhua11)/((baisha1016/A.villosa)/luhua9))-0-0-7-1	Yes	5.60%
41	2017-W154	yuhua15/((YZ9102/yuhua11)/((baisha1016/A.villosa)/luhua9))-0-0-72-1	Yes	9.30%
42	2017-W161	yuhua15/((YZ9102/yuhua11)/((baisha1016/A.villosa)/luhua9))-0-0-122-1	Yes	2.80%
43	2017-W165	yuhua15/((YZ9102/yuhua11)/((baisha1016/A.villosa)/luhua9))-0-0-188-1	Yes	0.00%
44	2017-W324	yuhua15/((YZ9102/yuhua11)/((baisha1016/A.vill)/luhua9))-0-0-sd-16	Yes	0.00%
45	luohua8	YZ9102/yuhua15	Yes	0.00%
46	yuhua40	9541-2-1/YZ9102	Yes	2.90%
47	yuanza5	YZ9102/shiyouhong4	Yes	11.80%
48	yuanza9614	YZ9102/yuhua11	Yes	20.80%
49	2015-W277	(baisha1016/(fuqing/A.chacoense))/(zheng8903/yuhua4)(w0827)-N-0-Sd-Sd-Sd-Sd-3	No	80.00%
50	2015-W279	(baisha1016/(fuqing/A.chacoense))/ ((baisha1016/A.villosa)/luhua9)(w0828)-N-0-Sd-Sd-Sd-Sd-4	No	98.90%
51	2015-W376	(baisha1016/(fuqing/A.chacoense))/m04-0213(w1007)-N1-0-83-1-1	No	95.00%
52	2017-W1111	huayu20/(86036/A.mon)(w0833)-N-/-0-Sd-Sd-Sd-2-1	No	70.50%
53	2017-W1210	yuhua9/mutant1 with high oil(w1103)-0-0-113	No	89.30%
54	2017-W1211	yuhua1/mutant1 with high protein(w1105)-0-0-57	No	82.20%
55	2017-W242	(baisha1016/(fuqing/A.chacoense))/(baisha1016/(zheng8903/yuhua4))(w1016)-0-0-sd-sd-25	No	71.20%
56	2017-W245	(baisha1016/(fuqing/A.chacoense))/(baisha1016/(zheng8903/yuhua4))(w1016)-0-0-sd-sd-48	No	73.30%
57	2017-W297	yuhua9/mutant with high oil(w1103)-0-0-sd-13	No	72.20%
58	2017-W268	(baisha1016/(fuqing/A.chacoense))/(baisha1016/kainongxuan01-6）(w1020)-0-0-sd-sd-25	No	8.50%
59	2017-W294	yuhua1/w4296(w1106)-0-0-sd-sd-46	No	10.80%
60	2017-W295	yuhua1/w4296(w1106)-0-0-sd-sd-72	No	21.00%
61	2017-W201	(baisha1016/(fuqing/A.chacoense))/wt08-0932(w1006)-N1-0-Sd-sd-sd-3	No	4.80%
62	2017-W296	yuhua9/mutant1 with high oil(w1103)-0-0-sd-6	No	44.30%
63	2015-W272	yuhua9502/(yuhua7/(fuqing/(lankaoduoli/A.chacoense)))(w0825)-N-0-Sd-Sd-Sd-Sd-10	No	33.80%
64	2017-W1002	yuhua9326/(86036/A.mon)(w0822)-N-0-Sd-Sd-Sd-6-1-1	No	0.00%
65	2017-W1003	yuhua9326/(86036/A.mon)(w0822)-N-0-Sd-Sd-Sd-6-1-2	No	0.00%
66	2017-W1004	yuhua9326/(86036/A.mon)(w0822)-N-0-Sd-Sd-Sd-1-2	No	0.00%
67	2017-W1008	(baisha1016/(fuqing/A.chacoense))/(baisha1016/kainongxuan01-6）(w1020)-0-0-23-1-1	No	0.00%
68	2017-W107	jinhua5/(yuhua4/(A.chacoense/(xihuaxiaozibai/A.chacoense)))(w0724)-0-0-Sd-Sd-Sd-Sd-6-1-1	No	0.00%
69	2017-W1106	jinhua5/(yuhua4/(A.chacoense/(xihuaxiaozibai/A.chacoense)))(w0724)-0-0-Sd-Sd-Sd-Sd-5-1	No	0.00%
70	2017-W1309	baisha1016/A.monticola(W0603)-N-0-23-Sd(N)-Sd-Sd-Sd-1(N)-1-B-B	No	5.60%
71	2017-W302	yuhua9/mutant1 with high oil(w1103)-0-0-sd-39	No	15.70%
72	2017-W313	yuhua9326/mutant1 with high oleic acid (w1104)-0-0-sd-56	No	0.00%

Supplementary Table S6 Bacterial wilt resistance of 317 peanut germplasms

ID	Variant	Survival Rate-2019 %	Survival Rate-2020 %
N730	*A. hypogaea* subsp. *fastigata* var. *vulgaris*	80.00%	86.36%
N853	*A. hypogaea* subsp. *fastigata* var. *vulgaris*	100.00%	90.63%
N551	*A. hypogaea* subsp. *fastigata* var. *vulgaris*	100.00%	75.00%
N495	*A. hypogaea* subsp. *fastigata* var. *vulgaris*	100.00%	85.78%
N425	*A. hypogaea* subsp. *fastigata* var. *vulgaris*	100.00%	85.13%
N725	*A. hypogaea* subsp. *fastigata* var. *vulgaris*	100.00%	93.30%
N695	*A. hypogaea* subsp. *fastigata* var. *vulgaris*	100.00%	96.43%
N412	*A. hypogaea* subsp. *fastigata* var. *vulgaris*	100.00%	86.67%
N453	*A. hypogaea* subsp. *fastigata* var. *vulgaris*	100.00%	84.96%
N406	*A. hypogaea* subsp. *fastigata* var. *vulgaris*	100.00%	71.05%
N523	*A. hypogaea* subsp. *fastigata* var. *vulgaris*	100.00%	76.67%
N498	*A. hypogaea* subsp. *fastigata* var. *vulgaris*	95.00%	97.06%
N609	*A. hypogaea* subsp. *fastigata* var. *vulgaris*	94.74%	70.38%
N610	*A. hypogaea* subsp. *fastigata* var. *fastigiata*	94.44%	93.75%
N590	*A. hypogaea* subsp. *fastigata* var. *vulgaris*	94.12%	89.01%
N414	*A. hypogaea* subsp. *fastigata* var. *vulgaris*	94.12%	96.43%
N537	*A. hypogaea* subsp. *fastigata* var. *vulgaris*	94.12%	72.22%
N756	*A. hypogaea* subsp. *fastigata* var. *vulgaris*	93.75%	92.68%
N575	*A. hypogaea* subsp. *fastigata* var. *vulgaris*	90.00%	77.47%
N711	*A. hypogaea* subsp. *fastigata* var. *vulgaris*	89.47%	84.03%
N554	*A. hypogaea* subsp. *fastigata* var. *vulgaris*	89.47%	46.41%
N420	*A. hypogaea* subsp. *fastigata* var. *vulgaris*	89.47%	72.22%
N716	*A. hypogaea* subsp. *fastigata* var. *vulgaris*	88.89%	95.00%
N418	*A. hypogaea* subsp. *fastigata* var. *vulgaris*	88.89%	83.33%
N753	*A. hypogaea* subsp. *fastigata* var. *vulgaris*	85.71%	86.67%
N484	*A. hypogaea* subsp. *fastigata* var. *vulgaris*	85.71%	86.15%
N415	*A. hypogaea* subsp. *fastigata* var. *vulgaris*	85.71%	16.67%
N752	*A. hypogaea* subsp. *fastigata* var. *vulgaris*	83.33%	73.33%
N514	*A. hypogaea* subsp. *fastigata* var. *vulgaris*	80.00%	51.67%
N570	*A. hypogaea* subsp. *fastigata* var. *vulgaris*	80.00%	78.75%
N535	*A. hypogaea* subsp. *fastigata* var. *vulgaris*	80.00%	85.71%
N728	*A. hypogaea* subsp. *fastigata* var. *vulgaris*	80.00%	84.38%
N531	*A. hypogaea* subsp. *fastigata* var. *vulgaris*	75.00%	97.22%
N424	*A. hypogaea* subsp. *fastigata* var. *vulgaris*	75.00%	83.33%
N486	*A. hypogaea* subsp. *fastigata* var. *vulgaris*	73.68%	90.00%
N692	*A. hypogaea* subsp. *fastigata* var. *vulgaris*	21.43%	19.87%
N401	*A. hypogaea* subsp. *fastigata* var. *vulgaris*	50.00%	29.41%
N461	*A. hypogaea* subsp. *fastigata* var. *vulgaris*	57.14%	6.67%
N439	*A. hypogaea* subsp. *fastigata* var. *vulgaris*	14.29%	51.89%
N550	*A. hypogaea* subsp. *fastigata* var. *vulgaris*	18.75%	58.33%
N456	*A. hypogaea* subsp. *fastigata* var. *vulgaris*	27.78%	16.23%
N474	*A. hypogaea* subsp. *fastigata* var. *vulgaris*	11.76%	6.70%
N405	*A. hypogaea* subsp. *fastigata* var. *vulgaris*	30.00%	14.29%
N404	*A. hypogaea* subsp. *fastigata* var. *vulgaris*	43.75%	11.54%
N477	*A. hypogaea* subsp. *fastigata* var. *vulgaris*	11.76%	24.68%
N576	*A. hypogaea* subsp. *fastigata* var. *vulgaris*	43.75%	14.36%
N471	*A. hypogaea* subsp. *fastigata* var. *vulgaris*	43.75%	11.11%

ID	Variant	Survival Rate-2019 %	Survival Rate-2020 %
N448	*A. hypogaea* subsp. *fastigata* var. *vulgaris*	27.78%	22.73%
N466	*A. hypogaea* subsp. *fastigata* var. *vulgaris*	11.76%	10.71%
N553	*A. hypogaea* subsp. *fastigata* var. *vulgaris*	5.88%	16.67%
N442	*A. hypogaea* subsp. *fastigata* var. *vulgaris*	27.78%	3.85%
N593	*A. hypogaea* subsp. *fastigata* var. *vulgaris*	29.41%	25.89%
N559	*A. hypogaea* subsp. *fastigata* var. *vulgaris*	46.15%	8.33%
N530	*A. hypogaea* subsp. *fastigata* var. *vulgaris*	28.57%	20.83%
N601	*A. hypogaea* subsp. *fastigata* var. *vulgaris*	16.67%	5.00%
N457	*A. hypogaea* subsp. *fastigata* var. *vulgaris*	11.76%	21.59%
N413	*A. hypogaea* subsp. *fastigata* var. *vulgaris*	16.67%	7.14%
N568	*A. hypogaea* subsp. *fastigata* var. *vulgaris*	31.58%	16.67%
N449	*A. hypogaea* subsp. *fastigata* var. *vulgaris*	16.67%	19.64%
N547	*A. hypogaea* subsp. *fastigata* var. *vulgaris*	42.86%	33.08%
N569	*A. hypogaea* subsp. *fastigata* var. *vulgaris*	33.33%	28.57%
N469	*A. hypogaea* subsp. *fastigata* var. *vulgaris*	64.29%	61.11%
N742	*A. hypogaea* subsp. *fastigata* var. *vulgaris*	6.25%	10.56%
N524	*A. hypogaea* subsp. *fastigata* var. *vulgaris*	26.32%	6.25%
N727	*A. hypogaea* subsp. *fastigata* var. *vulgaris*	7.69%	29.86%
N561	*A. hypogaea* subsp. *fastigata* var. *vulgaris*	31.25%	20.59%
N464	*A. hypogaea* subsp. *fastigata* var. *vulgaris*	31.25%	12.92%
N472	*A. hypogaea* subsp. *fastigata* var. *vulgaris*	41.18%	5.56%
N712	*A. hypogaea* subsp. *fastigata* var. *vulgaris*	37.50%	25.00%
N422	*A. hypogaea* subsp. *fastigata* var. *vulgaris*	23.53%	45.38%
N732	*A. hypogaea* subsp. *fastigata* var. *vulgaris*	41.18%	24.36%
N467	*A. hypogaea* subsp. *fastigata* var. *vulgaris*	31.25%	11.54%
N410	*A. hypogaea* subsp. *fastigata* var. *vulgaris*	23.53%	13.94%
N432	*A. hypogaea* subsp. *fastigata* var. *vulgaris*	31.25%	40.97%
N417	*A. hypogaea* subsp. *fastigata* var. *vulgaris*	55.00%	35.00%
N437	*A. hypogaea* subsp. *fastigata* var. *vulgaris*	40.00%	35.83%
N429	*A. hypogaea* subsp. *fastigata* var. *vulgaris*	0.00%	19.01%
N476	*A. hypogaea* subsp. *fastigata* var. *vulgaris*	66.67%	27.23%
N460	*A. hypogaea* subsp. *fastigata* var. *vulgaris*	28.57%	51.25%
N485	*A. hypogaea* subsp. *fastigata* var. *vulgaris*	23.53%	39.39%
N708	*A. hypogaea* subsp. *fastigata* var. *vulgaris*	43.75%	60.00%
N822	*A. hypogaea* subsp. *fastigata* var. *vulgaris*	37.50%	41.43%
N427	*A. hypogaea* subsp. *fastigata* var. *vulgaris*	9.09%	64.29%
N706	*A. hypogaea* subsp. *fastigata* var. *vulgaris*	46.67%	26.67%
N431	*A. hypogaea* subsp. *fastigata* var. *fastigiata*	37.50%	0.00%
N421	*A. hypogaea* subsp. *fastigata* var. *fastigiata*	47.06%	10.00%
N462	*A. hypogaea* subsp. *fastigata* var. *fastigiata*	6.67%	18.68%
N411	*A. hypogaea* subsp. *fastigata* var. *fastigiata*	6.25%	4.17%
N544	*A. hypogaea* subsp. *fastigata* var. *fastigiata*	70.00%	3.57%
N724	*A. hypogaea* subsp. *hypogaea* var. *hypogaea*	18.75%	33.33%
N717	*A. hypogaea* subsp. *hypogaea* var. *hypogaea*	23.08%	27.43%
N526	*A. hypogaea* subsp. *hypogaea* var. *hypogaea*	23.53%	4.17%
N622	*A. hypogaea* subsp. *fastigata* var. *fastigiata*	62.50%	0.00%
N402	*A. hypogaea* subsp. *fastigata* var. *fastigiata*	47.37%	10.71%
N483	*A. hypogaea* subsp. *fastigata* var. *fastigiata*	64.71%	12.50%
N613	*A. hypogaea* subsp. *fastigata* var. *fastigiata*	41.18%	0.00%

ID	Variant	Survival Rate-2019 %	Survival Rate-2020 %
N451	*A. hypogaea* subsp. *fastigata* var. *fastigiata*	27.78%	9.40%
N549	*A. hypogaea* subsp. *fastigata* var. *fastigiata*	20.00%	15.38%
N618	*A. hypogaea* subsp. *fastigata* var. *fastigiata*	37.50%	7.69%
N548	*A. hypogaea* subsp. *fastigata* var. *fastigiata*	45.00%	10.71%
N479	*A. hypogaea* subsp. *fastigata* var. *fastigiata*	41.18%	17.42%
N438	*A. hypogaea* subsp. *fastigata* var. *fastigiata*	55.56%	20.19%
N608	*A. hypogaea* subsp. *fastigata* var. *fastigiata*	29.41%	12.14%
N468	*A. hypogaea* subsp. *fastigata* var. *fastigiata*	30.00%	7.74%
N543	*A. hypogaea* subsp. *fastigata* var. *fastigiata*	15.00%	17.19%
N434	*A. hypogaea* subsp. *fastigata* var. *fastigiata*	0.00%	9.09%
N435	*A. hypogaea* subsp. *fastigata* var. *fastigiata*	36.84%	0.00%
N455	*A. hypogaea* subsp. *fastigata* var. *fastigiata*	15.38%	13.33%
N505	*A. hypogaea* subsp. *fastigata* var. *fastigiata*	22.22%	3.33%
N564	*A. hypogaea* subsp. *fastigata* var. *fastigiata*	38.89%	11.54%
N589	*A. hypogaea* subsp. *fastigata* var. *fastigiata*	20.00%	7.14%
N741	*A. hypogaea* subsp. *fastigata* var. *fastigiata*	28.57%	0.00%
N450	*A. hypogaea* subsp. *fastigata* var. *vulgaris*	40.00%	20.51%
N443	*A. hypogaea* subsp. *fastigata* var. *vulgaris*	17.65%	7.69%
N563	*A. hypogaea* subsp. *hypogaea* var. *hirsuta*	6.67%	27.38%
N532	*A. hypogaea* subsp. *hypogaea* var. *hirsuta*	38.89%	11.76%
N675	*A. hypogaea* subsp. *hypogaea* var. *hirsuta*	36.84%	7.29%
N507	*A. hypogaea* subsp. *hypogaea* var. *hirsuta*	50.00%	5.56%
N614	*A. hypogaea* subsp. *hypogaea* var. *hirsuta*	36.84%	20.00%
N506	*A. hypogaea* subsp. *hypogaea* var. *hirsuta*	26.67%	38.57%
N625	*A. hypogaea* subsp. *hypogaea* var. *hirsuta*	35.71%	22.73%
N491	*A. hypogaea* subsp. *hypogaea* var. *hirsuta*	11.76%	61.22%
N665	*A. hypogaea* subsp. *hypogaea* var. *hirsuta*	5.88%	15.34%
N624	*A. hypogaea* subsp. *hypogaea* var. *hirsuta*	55.56%	15.34%
N644	*A. hypogaea* subsp. *hypogaea* var. *hirsuta*	18.75%	7.42%
N770	*A. hypogaea* subsp. *hypogaea* var. *hypogaea*	22.22%	0.00%
N739	*A. hypogaea* subsp. *hypogaea* var. *hypogaea*	60.00%	40.06%
N815	*A. hypogaea* subsp. *hypogaea* var. *hypogaea*	15.38%	65.00%
N541	*A. hypogaea* subsp. *hypogaea* var. *hypogaea*	37.50%	46.15%
N579	*A. hypogaea* subsp. *hypogaea* var. *hypogaea*	31.58%	17.86%
N689	*A. hypogaea* subsp. *hypogaea* var. *hypogaea*	20.00%	16.67%
N446	*A. hypogaea* subsp. *hypogaea* var. *hypogaea*	50.00%	18.45%
N581	*A. hypogaea* subsp. *hypogaea* var. *hypogaea*	55.56%	58.33%
N574	*A. hypogaea* subsp. *hypogaea* var. *hypogaea*	23.53%	5.56%
N534	*A. hypogaea* subsp. *hypogaea* var. *hypogaea*	42.86%	35.71%
N562	*A. hypogaea* subsp. *hypogaea* var. *hypogaea*	40.00%	30.15%
N661	*A. hypogaea* subsp. *hypogaea* var. *hypogaea*	37.50%	18.75%
N840	*A. hypogaea* subsp. *hypogaea* var. *hypogaea*	20.00%	50.00%
N510	*A. hypogaea* subsp. *hypogaea* var. *hypogaea*	33.33%	14.29%
N628	*A. hypogaea* subsp. *hypogaea* var. *hypogaea*	44.44%	23.61%
N620	*A. hypogaea* subsp. *hypogaea* var. *hypogaea*	21.05%	13.89%
N627	*A. hypogaea* subsp. *hypogaea* var. *hypogaea*	26.32%	23.64%
N744	*A. hypogaea* subsp. *hypogaea* var. *hypogaea*	42.86%	11.31%
N677	*A. hypogaea* subsp. *hypogaea* var. *hypogaea*	6.67%	11.86%
N556	*A. hypogaea* subsp. *hypogaea* var. *hypogaea*	18.75%	17.46%

ID	Variant	Survival Rate-2019 %	Survival Rate-2020 %
N518	*A. hypogaea* subsp. *hypogaea* var. *hypogaea*	44.44%	37.50%
N651	*A. hypogaea* subsp. *hypogaea* var. *hypogaea*	5.56%	24.04%
N445	*A. hypogaea* subsp. *hypogaea* var. *hypogaea*	31.58%	10.00%
N555	*A. hypogaea* subsp. *hypogaea* var. *hypogaea*	10.53%	25.00%
N691	*A. hypogaea* subsp. *hypogaea* var. *hypogaea*	31.25%	51.89%
N525	*A. hypogaea* subsp. *hypogaea* var. *hypogaea*	27.27%	16.67%
N540	*A. hypogaea* subsp. *hypogaea* var. *hypogaea*	18.75%	2.94%
N542	*A. hypogaea* subsp. *hypogaea* var. *hypogaea*	33.33%	20.00%
N566	*A. hypogaea* subsp. *hypogaea* var. *hypogaea*	20.00%	49.52%
N580	*A. hypogaea* subsp. *hypogaea* var. *hypogaea*	0.00%	13.33%
N591	*A. hypogaea* subsp. *hypogaea* var. *hypogaea*	11.76%	15.63%
N660	*A. hypogaea* subsp. *hypogaea* var. *hypogaea*	18.75%	3.57%
N668	*A. hypogaea* subsp. *hypogaea* var. *hypogaea*	11.76%	8.82%
N671	*A. hypogaea* subsp. *hypogaea* var. *hypogaea*	18.75%	20.78%
N721	*A. hypogaea* subsp. *hypogaea* var. *hypogaea*	21.43%	43.59%
N743	*A. hypogaea* subsp. *hypogaea* var. *hypogaea*	7.14%	0.00%
N767	*A. hypogaea* subsp. *hypogaea* var. *hypogaea*	23.53%	12.14%
N854	*A. hypogaea* subsp. *hypogaea* var. *hypogaea*	20.00%	59.38%
N546	*A. hypogaea* subsp. *hypogaea* var. *hypogaea*	50.00%	58.65%
N664	*A. hypogaea* subsp. *hypogaea* var. *hypogaea*	41.18%	6.25%
N816	*A. hypogaea* subsp. *hypogaea* var. *hypogaea*	50.00%	49.39%
N487	*A. hypogaea* subsp. *hypogaea* var. *hypogaea*	10.53%	7.69%
N516	*A. hypogaea* subsp. *hypogaea* var. *hypogaea*	16.67%	45.71%
N538	*A. hypogaea* subsp. *hypogaea* var. *hypogaea*	44.44%	36.61%
N843	*A. hypogaea* subsp. *hypogaea* var. *hypogaea*	16.67%	36.32%
N847	*A. hypogaea* subsp. *hypogaea* var. *hypogaea*	10.53%	57.29%
N647	*A. hypogaea* subsp. *hypogaea* var. *hypogaea*	40.00%	55.21%
N656	*A. hypogaea* subsp. *hypogaea* var. *hypogaea*	35.29%	11.11%
N642	*A. hypogaea* subsp. *hypogaea* var. *hypogaea*	17.65%	27.27%
N621	*A. hypogaea* subsp. *hypogaea* var. *hypogaea*	33.33%	18.82%
N638	*A. hypogaea* subsp. *hypogaea* var. *hypogaea*	10.53%	34.92%
N645	*A. hypogaea* subsp. *hypogaea* var. *hypogaea*	15.38%	18.18%
N779	*A. hypogaea* subsp. *hypogaea* var. *hypogaea*	22.22%	15.63%
N496	*A. hypogaea* subsp. *hypogaea* var. *hypogaea*	13.33%	35.00%
N650	*A. hypogaea* subsp. *hypogaea* var. *hypogaea*	20.00%	17.42%
N652	*A. hypogaea* subsp. *hypogaea* var. *hypogaea*	26.67%	4.55%
N842	*A. hypogaea* subsp. *hypogaea* var. *hypogaea*	6.67%	10.80%
N851	*A. hypogaea* subsp. *hypogaea* var. *hypogaea*	13.33%	43.81%
N637	*A. hypogaea* subsp. *hypogaea* var. *hypogaea*	12.50%	10.24%
N735	*A. hypogaea* subsp. *hypogaea* var. *hypogaea*	56.25%	51.37%
N557	*A. hypogaea* subsp. *hypogaea* var. *hypogaea*	16.67%	31.92%
N635	*A. hypogaea* subsp. *hypogaea* var. *hypogaea*	11.11%	14.71%
N768	*A. hypogaea* subsp. *hypogaea* var. *hypogaea*	16.67%	33.33%
N646	*A. hypogaea* subsp. *hypogaea* var. *hypogaea*	35.29%	14.24%
N482	*A. hypogaea* subsp. *hypogaea* var. *hypogaea*	33.33%	18.75%
N492	*A. hypogaea* subsp. *hypogaea* var. *hypogaea*	11.76%	17.86%
N508	*A. hypogaea* subsp. *hypogaea* var. *hypogaea*	50.00%	7.14%
N539	*A. hypogaea* subsp. *hypogaea* var. *hypogaea*	18.75%	17.18%
N558	*A. hypogaea* subsp. *hypogaea* var. *hypogaea*	44.44%	53.13%

ID	Variant	Survival Rate-2019 %	Survival Rate-2020 %
N634	*A. hypogaea* subsp. *hypogaea* var. *hypogaea*	17.65%	7.69%
N653	*A. hypogaea* subsp. *hypogaea* var. *hypogaea*	41.18%	5.00%
N667	*A. hypogaea* subsp. *hypogaea* var. *hypogaea*	5.26%	7.14%
N672	*A. hypogaea* subsp. *hypogaea* var. *hypogaea*	26.67%	19.17%
N713	*A. hypogaea* subsp. *hypogaea* var. *hypogaea*	20.00%	23.53%
N490	*A. hypogaea* subsp. *hypogaea* var. *hypogaea*	30.77%	55.95%
N707	*A. hypogaea* subsp. *hypogaea* var. *hypogaea*	38.89%	13.94%
N818	*A. hypogaea* subsp. *hypogaea* var. *hypogaea*	26.32%	16.67%
N536	*A. hypogaea* subsp. *hypogaea* var. *hypogaea*	57.89%	0.00%
N699	*A. hypogaea* subsp. *hypogaea* var. *hypogaea*	30.00%	29.17%
N577	*A. hypogaea* subsp. *hypogaea* var. *hypogaea*	22.22%	13.33%
N702	*A. hypogaea* subsp. *hypogaea* var. *hypogaea*	27.78%	59.19%
N545	*A. hypogaea* subsp. *hypogaea* var. *hypogaea*	61.11%	41.67%
N700	*A. hypogaea* subsp. *hypogaea* var. *hypogaea*	50.00%	5.00%
N408	*A. hypogaea* subsp. *hypogaea* var. *hypogaea*	25.00%	37.50%
N470	*A. hypogaea* subsp. *hypogaea* var. *hypogaea*	26.32%	40.38%
N604	*A. hypogaea* subsp. *hypogaea* var. *hypogaea*	52.63%	12.50%
N696	*A. hypogaea* subsp. *hypogaea* var. *hypogaea*	26.32%	22.50%
N850	*A. hypogaea* subsp. *hypogaea* var. *hypogaea*	38.46%	3.13%
N789	*A. hypogaea* subsp. *hypogaea* var. *hypogaea*	18.18%	31.41%
N606	*A. hypogaea* subsp. *hypogaea* var. *hypogaea*	20.00%	37.01%
N503	*A. hypogaea* subsp. *hypogaea* var. *hypogaea*	42.86%	29.87%
N595	*A. hypogaea* subsp. *hypogaea* var. *hypogaea*	14.29%	48.33%
N790	*A. hypogaea* subsp. *hypogaea* var. *hypogaea*	41.18%	35.00%
N762	*A. hypogaea* subsp. *hypogaea* var. *hypogaea*	25.00%	18.75%
N585	*A. hypogaea* subsp. *hypogaea* var. *hypogaea*	16.67%	21.49%
N693	*A. hypogaea* subsp. *hypogaea* var. *hypogaea*	8.33%	27.88%
N489	*A. hypogaea* subsp. *hypogaea* var. *hypogaea*	7.69%	3.85%
N750	*A. hypogaea* subsp. *hypogaea* var. *hypogaea*	28.57%	22.22%
N718	*A. hypogaea* subsp. *hypogaea* var. *hypogaea*	33.33%	43.18%
N765	*A. hypogaea* subsp. *hypogaea* var. *hypogaea*	46.67%	38.89%
N502	*A. hypogaea* subsp. *hypogaea* var. *hypogaea*	6.25%	0.00%
N838	*A. hypogaea* subsp. *hypogaea* var. *hypogaea*	25.00%	13.39%
N499	*A. hypogaea* subsp. *hypogaea* var. *hypogaea*	26.32%	8.33%
N407	*A. hypogaea* subsp. *hypogaea* var. *hypogaea*	26.32%	32.74%
N501	*A. hypogaea* subsp. *hypogaea* var. *hypogaea*	41.18%	20.00%
N511	*A. hypogaea* subsp. *hypogaea* var. *hypogaea*	0.00%	13.84%
N528	*A. hypogaea* subsp. *hypogaea* var. *hypogaea*	7.14%	10.00%
N701	*A. hypogaea* subsp. *hypogaea* var. *hypogaea*	37.50%	54.76%
N703	*A. hypogaea* subsp. *hypogaea* var. *hypogaea*	6.67%	18.83%
N719	*A. hypogaea* subsp. *hypogaea* var. *hypogaea*	26.67%	25.48%
N734	*A. hypogaea* subsp. *hypogaea* var. *hypogaea*	35.29%	44.64%
N754	*A. hypogaea* subsp. *hypogaea* var. *hypogaea*	16.67%	13.49%
N630	*A. hypogaea* subsp. *hypogaea* var. *hypogaea*	29.41%	28.17%
N710	*A. hypogaea* subsp. *hypogaea* var. *hypogaea*	28.57%	48.35%
N473	*A. hypogaea* subsp. *hypogaea* var. *hypogaea*	40.00%	19.17%
N698	*A. hypogaea* subsp. *hypogaea* var. *hypogaea*	40.00%	54.76%
N603	*A. hypogaea* subsp. *hypogaea* var. *hypogaea*	57.14%	6.67%
N731	*A. hypogaea* subsp. *hypogaea* var. *hypogaea*	35.29%	50.59%

ID	Variant	Survival Rate-2019 %	Survival Rate-2020 %
N705	*A. hypogaea* subsp. *hypogaea* var. *hypogaea*	27.78%	22.25%
N600	*A. hypogaea* subsp. *hypogaea* var. *hypogaea*	35.71%	26.44%
N594	*A. hypogaea* subsp. *hypogaea* var. *hypogaea*	18.75%	18.61%
N632	*A. hypogaea* subsp. *hypogaea* var. *hypogaea*	20.00%	6.46%
N776	*A. hypogaea* subsp. *hypogaea* var. *hypogaea*	25.00%	19.09%
N598	*A. hypogaea* subsp. *hypogaea* var. *hypogaea*	47.06%	8.82%
N666	*A. hypogaea* subsp. *hypogaea* var. *hypogaea*	47.06%	19.64%
N766	*A. hypogaea* subsp. *hypogaea* var. *hypogaea*	14.29%	7.14%
N433	*A. hypogaea* subsp. *hypogaea* var. *hypogaea*	31.58%	19.19%
N481	*A. hypogaea* subsp. *hypogaea* var. *hypogaea*	26.32%	11.76%
N428	*A. hypogaea* subsp. *hypogaea* var. *hypogaea*	35.29%	57.34%
N636	*A. hypogaea* subsp. *hypogaea* var. *hypogaea*	29.41%	10.99%
N640	*A. hypogaea* subsp. *hypogaea* var. *hypogaea*	10.00%	14.05%
N723	*A. hypogaea* subsp. *hypogaea* var. *hypogaea*	16.67%	20.00%
N674	*A. hypogaea* subsp. *hypogaea* var. *hypogaea*	46.15%	45.78%
N715	*A. hypogaea* subsp. *hypogaea* var. *hypogaea*	35.29%	40.98%
N720	*A. hypogaea* subsp. *hypogaea* var. *hypogaea*	11.11%	34.24%
N633	*A. hypogaea* subsp. *hypogaea* var. *hypogaea*	46.67%	19.55%
N648	*A. hypogaea* subsp. *hypogaea* var. *hypogaea*	33.33%	19.17%
N626	*A. hypogaea* subsp. *hypogaea* var. *hypogaea*	17.65%	5.56%
N509	*A. hypogaea* subsp. *hypogaea* var. *hypogaea*	18.18%	41.96%
N521	*A. hypogaea* subsp. *hypogaea* var. *hypogaea*	10.53%	48.21%
N684	*A. hypogaea* subsp. *hypogaea* var. *hypogaea*	20.00%	18.18%
N527	*A. hypogaea* subsp. *hypogaea* var. *hypogaea*	25.00%	28.08%
N619	*A. hypogaea* subsp. *hypogaea* var. *hypogaea*	7.14%	3.57%
N764	*A. hypogaea* subsp. *hypogaea* var. *hypogaea*	33.33%	12.50%
N722	*A. hypogaea* subsp. *hypogaea* var. *hypogaea*	7.14%	20.83%
N629	*A. hypogaea* subsp. *hypogaea* var. *hypogaea*	37.50%	20.98%
N641	*A. hypogaea* subsp. *hypogaea* var. *hypogaea*	6.25%	17.18%
N729	*A. hypogaea* subsp. *hypogaea* var. *hypogaea*	21.05%	10.00%
N605	*A. hypogaea* subsp. *hypogaea* var. *hypogaea*	25.00%	52.78%
N631	*A. hypogaea* subsp. *hypogaea* var. *hypogaea*	18.75%	8.33%
N662	*A. hypogaea* subsp. *hypogaea* var. *hypogaea*	27.78%	18.96%
N685	*A. hypogaea* subsp. *hypogaea* var. *hypogaea*	26.32%	0.00%
N687	*A. hypogaea* subsp. *hypogaea* var. *hypogaea*	26.32%	35.00%
N616	*A. hypogaea* subsp. *hypogaea* var. *hypogaea*	30.00%	12.50%
N512	*A. hypogaea* subsp. *hypogaea* var. *hypogaea*	7.69%	19.46%
N607	*A. hypogaea* subsp. *hypogaea* var. *hypogaea*	8.33%	17.14%
N623	*A. hypogaea* subsp. *hypogaea* var. *hypogaea*	17.65%	33.81%
N639	*A. hypogaea* subsp. *hypogaea* var. *hypogaea*	33.33%	4.55%
N649	*A. hypogaea* subsp. *hypogaea* var. *hypogaea*	27.78%	22.05%
N658	*A. hypogaea* subsp. *hypogaea* var. *hypogaea*	30.77%	16.59%
N669	*A. hypogaea* subsp. *hypogaea* var. *hypogaea*	0.00%	6.67%
N670	*A. hypogaea* subsp. *hypogaea* var. *hypogaea*	15.79%	16.67%
N673	*A. hypogaea* subsp. *hypogaea* var. *hypogaea*	15.79%	11.67%
N704	*A. hypogaea* subsp. *hypogaea* var. *hypogaea*	16.67%	6.90%
N738	*A. hypogaea* subsp. *hypogaea* var. *hypogaea*	5.88%	61.11%
N830	*A. hypogaea* subsp. *hypogaea* var. *hypogaea*	23.53%	31.90%
N834	*A. hypogaea* subsp. *hypogaea* var. *hypogaea*	22.22%	21.05%

ID	Variant	Survival Rate-2019 %	Survival Rate-2020 %
N849	*A. hypogaea* subsp. *hypogaea* var. *hypogaea*	31.25%	21.11%
N493	*A. hypogaea* subsp. *fastigata* var. *vulgaris*	54.55%	38.89%
N436	*A. hypogaea* subsp. *fastigata* var. *vulgaris*	27.78%	14.29%
N586	*A. hypogaea* subsp. *fastigata* var. *vulgaris*	41.18%	13.64%
N856	*A. hypogaea* subsp. *fastigata* var. *vulgaris*	26.32%	29.23%
N688	*A. hypogaea* subsp. *fastigata* var. *vulgaris*	16.67%	18.75%
N444	*A. hypogaea* subsp. *fastigata* var. *vulgaris*	21.43%	17.69%
N475	*A. hypogaea* subsp. *fastigata* var. *vulgaris*	23.53%	0.00%
N447	*A. hypogaea* subsp. *fastigata* var. *vulgaris*	23.53%	29.41%
N478	*A. hypogaea* subsp. *fastigata* var. *vulgaris*	7.69%	28.75%
N567	*A. hypogaea* subsp. *fastigata* var. *vulgaris*	13.33%	11.76%
N565	*A. hypogaea* subsp. *fastigata* var. *vulgaris*	57.89%	16.67%
N686	*A. hypogaea* subsp. *fastigata* var. *vulgaris*	23.53%	16.31%
N615	*A. hypogaea* subsp. *fastigata* var. *vulgaris*	42.86%	4.17%
N465	*A. hypogaea* subsp. *fastigata* var. *vulgaris*	17.65%	31.60%
N463	*A. hypogaea* subsp. *fastigata* var. *vulgaris*	21.05%	34.38%
N855	*A. hypogaea* subsp. *fastigata* var. *vulgaris*	18.75%	18.75%
N419	*A. hypogaea* subsp. *fastigata* var. *vulgaris*	12.50%	50.00%
N714	*A. hypogaea* subsp. *fastigata* var. *vulgaris*	28.57%	7.69%
N697	*A. hypogaea* subsp. *fastigata* var. *vulgaris*	26.32%	31.92%
N497	*A. hypogaea* subsp. *fastigata* var. *vulgaris*	26.32%	26.14%
N748	*A. hypogaea* subsp. *fastigata* var. *vulgaris*	38.46%	43.75%
N751	*A. hypogaea* subsp. *fastigata* var. *vulgaris*	0.00%	0.00%
N848	*A. hypogaea* subsp. *fastigata* var. *vulgaris*	25.00%	58.18%
N852	*A. hypogaea* subsp. *fastigata* var. *vulgaris*	50.00%	59.38%
